# Periarteriolar spaces modulate cerebrospinal fluid transport into brain and demonstrate altered morphology in aging and Alzheimer’s disease

**DOI:** 10.1038/s41467-022-31257-9

**Published:** 2022-07-06

**Authors:** Humberto Mestre, Natasha Verma, Thom D. Greene, LiJing A. Lin, Antonio Ladron-de-Guevara, Amanda M. Sweeney, Guojun Liu, V. Kaye Thomas, Chad A. Galloway, Karen L. de Mesy Bentley, Maiken Nedergaard, Rupal I. Mehta

**Affiliations:** 1 Center for Translational Neuromedicine, University of Rochester Medical Center, Rochester, NY 14642 USA; 2 Department of Neuroscience, University of Rochester Medical Center, Rochester, NY 14642 USA; 3 Department of Neurology, University of Pennsylvania, Philadelphia, PA 19104 USA; 4 Department of Pharmacology and Physiology, University of Rochester Medical Center, Rochester, NY 14642 USA; 5 Department of Pathology, University of Rochester Medical Center, Rochester, NY 14642 USA; 6 Center for Translational Neuromedicine, Faculty of Health and Medical Sciences, University of Copenhagen, 2200 Copenhagen, Denmark; 7 Department of Pathology, Rush University Medical Center, Chicago, IL 60612 USA; 8 Rush Alzheimer’s Disease Center, Rush University Medical Center, Chicago, IL 60612 USA

**Keywords:** Diseases of the nervous system, Neuro-vascular interactions, Neurophysiology, Ageing

## Abstract

Perivascular spaces (PVS) drain brain waste metabolites, but their specific flow paths are debated. Meningeal pia mater reportedly forms the outermost boundary that confines flow around blood vessels. Yet, we show that pia is perforated and permissive to PVS fluid flow. Furthermore, we demonstrate that pia is comprised of vascular and cerebral layers that coalesce in variable patterns along leptomeningeal arteries, often merging around penetrating arterioles. Heterogeneous pial architectures form variable sieve-like structures that differentially influence cerebrospinal fluid (CSF) transport along PVS. The degree of pial coverage correlates with macrophage density and phagocytosis of CSF tracer. In vivo imaging confirms transpial influx of CSF tracer, suggesting a role of pia in CSF filtration, but not flow restriction. Additionally, pial layers atrophy with age. Old mice also exhibit areas of pial denudation that are not observed in young animals, but pia is unexpectedly hypertrophied in a mouse model of Alzheimer’s disease. Moreover, pial thickness correlates with improved CSF flow and reduced β-amyloid deposits in PVS of old mice. We show that PVS morphology in mice is variable and that the structure and function of pia suggests a previously unrecognized role in regulating CSF transport and amyloid clearance in aging and disease.

## Abstract (web summary)

The precise boundaries and flow compartments of perivascular spaces in the brain are incompletely understood. Here the authors show that pia is perforated and permissive to CSF flow, forming three types of perivascular spaces that remodel with age, with an abnormal type arising in Alzheimer’s disease and correlating with β-amyloid burden and differential macrophage uptake.

## Introduction

Cerebrospinal fluid (CSF) imparts neurorestorative functions, serving unique roles in development, immunity, and brain maintenance^1^. It exchanges with brain interstitial fluid (ISF) by traversing a brain-wide network of perivascular spaces (PVS)^2,3^. Notably, CSF–ISF exchange has been demonstrated to facilitate the clearance of metabolic brain waste, such as β-amyloid^4,5^. PVS are often regarded as *Virchow–Robin spaces* (VRS)^6^, yet the anatomy and boundaries of these spaces have never been clearly depicted. Indeed, original descriptions by Virchow and Robin disputed VRS structure^7,8^, although subsequent literature generally summarizes them as homogeneous perivascular reflections comprised of simple pial membranes^9,10^. Some have suggested that CSF crosses pial membranes to enter PVS by percolating through pial pores that localize to adventitia of leptomeningeal vessels^11,12^. However, others argue against the existence of PVS and the localization of associated pial pores^13^. Weller and colleagues maintain that pial membranes constitute impenetrable sheets that restrict fluid movement at the brain surface and hypothesize that CSF permeates along underlying pial–glial basement membranes^14,15^. According to Weller’s model, CSF enters pial deficiencies that are present in deep cortical brain regions, only^13^. Recently, in vivo two-photon imaging studies have reignited the debate into fluid pathways by illustrating in real-time perivascular entry of CSF into brains of live rodents, supported by astrocytes through the glymphatic (i.e., glial-lymphatic) system^16^. Since this discovery, revised work by Weller and colleagues suggest that CSF enters brain alongside a separate space, termed the *paravascular* space, while ISF drains from brain via the *perivascular* route that was previously described^14^. Still, others conclude that VRS/PVS are discontinuous with brain parenchyma, representing blind-ending pouches or *cul de sacs*^17^.

Whereas these theories form the bases for current PVS models, anatomic evidence remains limited and little work has been published since the time of early PVS descriptions^7,8,18^. Surprisingly few investigations have comprehensively and systematically evaluated the morphology of pia mater or the fluid pathways next to cerebral vessels, and discrepant reporting has resulted in an abundance of nomenclatures and partial anatomic descriptions. Given inconsistent interpretations, this study was undertaken with the aim of elucidating pial and PVS structure and tracer movement patterns at cerebral cortical surfaces in mice. Using high-resolution imaging incorporating labeling with ERTR7, a newly reported pial cell marker that is highly expressed in reticulin-rich stroma of various lymphoid organs^19^, we illustrate the reticulated structure of pial cells and show that they produce a fenestrated meshwork along murine brain and afferent leptomeningeal vascular surfaces. We further demonstrate that heterogeneous pial reflections create unique anatomy and partial subcompartments next to penetrating arterioles. Moreover, we show that small cisternal tracer readily crosses the pial layers to enter PVS and induces macrophagic responses that correlate with the density of the pial ensheathments. Additionally, we report attenuation of pia in PVS of old mice but expansion in age-matched APP/PS1 animals, a mouse model of Alzheimer’s disease (AD), in which its density inversely correlates with the burden of amyloid deposits.

This study provides a reappraisal of pial structure, depicting for the first time its heterogeneous and porous nature that notably recapitulates the stroma of secondary lymphoid organs. Thereby, this work fundamentally alters our understanding of meningeal and cerebrovascular anatomy and provides a framework for investigating and interpreting pial function and for understanding the mechanisms associated with cerebrovascular and brain diseases.

## Results

### Intimal pial cells are reticulated, lack a basement membrane and form a fenestrated sheath at the brain surface

**Fig. 1:**
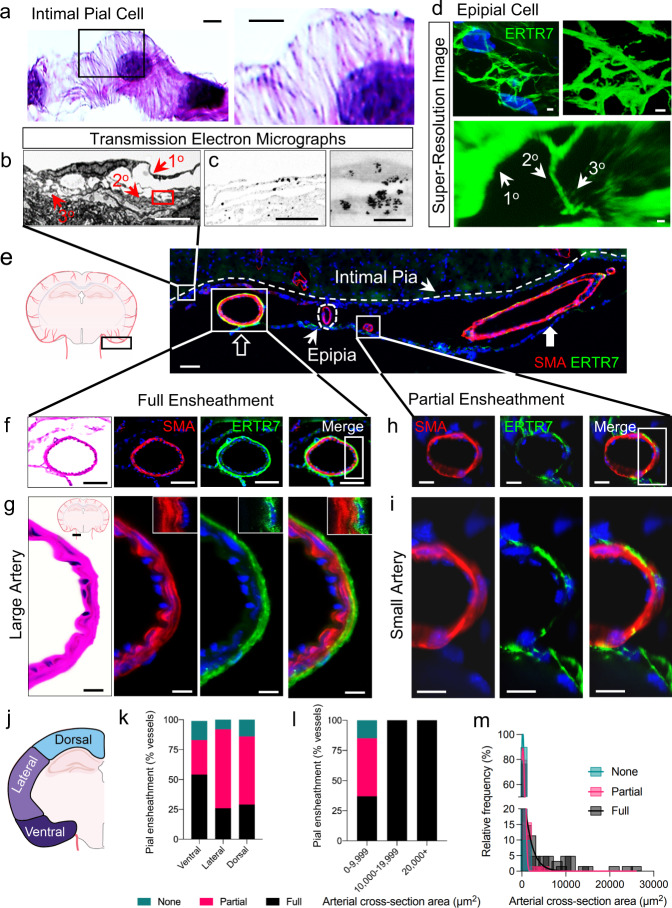
Pial cells are reticulated, express ERTR7, and prominently ensheath large arteries in ventral brain regions, forming the epipia. Routine (**a**), ultrastructural (**b**, **c**), and super-resolution fluorescent ERTR7 labeled (**d**) images of the brain surface demonstrate the reticulated nature of pial cells. On coronal section (**e**), the proximal middle cerebral artery is observed to be thickly ensheathed (open arrow), while a distal oblique arterial segment exhibits a thin sheath (solid arrow) and small arterial branches are unsheathed by ERTR7. Large subarachnoid arteries in ventral brain region are depicted on longitudinal (**e**) and cross (**e**, **f**) sections. Enlargement of the boxed area in **f** highlights ERTR7-positive tunica adventitia in apposition to the arterial tunica media (**g**); insets at upper right are representative of the boxed area and show punctate ERTR7 label, consistent with matted pial cell processes. Partial pial ensheathment is appreciated on cross section of a small artery (**h**), and enlargement of the boxed area (**i**). Diagram depicting brain regions used for quantification (**j**). Analysis of epipial coverage according to anatomical brain region (**k**) and vessel size (**l**, **m**); *n* = 145 vessels from 6 mice. **a** H&E; **b** transmission electron micrograph; **c** Immuno-EM for ERTR7; **d**–**i** green/FITC, ERTR7; red/CY3, SMA; blue, DAPI; scale bars = (**a**–**d**) 2 μm; **c** and **d** (insets) 200 nm; **e**, **f** 50 μm; **g**–**i** 10 μm. Primary (1^o^), secondary (2^o^), and tertiary (3^o^) processes are depicted within pial cells (**b**, **d**). Source data are provided as a Source Data file. TEM data are representative of 50 vessels from 3 young mice and immuno-EM data are representative of 6 sections from 2 young mice.

To understand pial anatomy, we first investigated the cytologic features of individual pial cells and discovered that this cell type is uniformly reticulated (Fig. 1a–d; Supplementary Fig. 1). H&E preparations demonstrate the reticulated nature of pial cells at the brain surface (Fig. 1a), where they constitute the *intimal pia*^6^. Ultrastructural imaging demonstrates that cells of this layer contain scant perinuclear cytoplasm from which elongated processes emanate (Fig. 1b; Supplementary Fig. 1). The pial cell processes display primary, secondary, and tertiary branches (Fig. 1b) that adjoin to adjacent processes and those of neighboring pial cells through intercellular junctions (i.e., tight junctions; Supplementary Fig. 1a). Cytosolic accumulations and scattered mitochondria produce irregular swellings that impart a beaded appearance within pial processes (Fig. 1c, Supplementary Fig. 1b, c). High-power micrographs reveal abundant intermediate filaments, ribosomes, and rough endoplasmic reticulum within the cytosol of intermitochondrial regions (Supplementary Fig. 1b, c). Fibrils of collagen are situated next to the pial cells (Fig. 1b, c, Supplementary Fig. 1b, c), however extracellular matrix is scant and pial basal laminae and/or basement membrane structure is not seen.

Given a recent report describing plectin and ERTR7 as potential pial cell markers^19^, we next validated anti-plectin and anti-ERTR7 antibodies but discovered that plectin co-localized with aquaporin-4 label within astrocyte foot processes^20^, while ERTR7 diffusely labeled pial cells at the brain surfaces (Supplementary Fig. 2). Thus, anti-ERTR7 antibodies were used to further investigate pial histology which is obscure on routine staining. Immuno-EM (Fig. 1b) and immunofluorescent (Fig. 1c; Supplementary Fig. 1d–g) labeling demonstrated that ERTR7 diffusely highlights the cytosol of intimal pial cells. High-power confocal and super-resolution microscopy of immunofluorescent-labeled brain sections were found to outline the pial cell processes as fine, branching, piloid elements with beaded appearance (Supplementary Fig.  1d–g), thus corroborating the light and transmission electron microscopic (TEM) appearances (Fig. 1b, c; Supplementary Fig. 1a–c). Using multiplanar imaging, we demonstrate that an interconnected pial cell meshwork loosely coats the brain surfaces, producing a complex superficial cell web (Supplementary Fig. 1d–g). While immunohistochemistry briskly labeled the pial elements, microscopic areas devoid of ERTR7 label were readily visible within this meshwork, constituting microfenestrae within the interwoven pial cell sheath (Supplementary Fig. 1d–g).

### Epipial cells are reticulated and form a dense sheath that comprises the tunica adventitia on primary cerebral arteries

To understand the anatomy of pia around the brain arteries, we next investigated perivascular ERTR7 label and discovered that in addition to highlighting the intimal pia, brisk ERTR7 label is present around primary cerebral arteries. In this location, the pial ensheathment constitutes the *epipia* (Fig. 1d–g)^6^. The cytologic appearances of pial cells within this layer is found to be identical to that of the intimal pia. Super-resolution images of an epipial cell are depicted in Fig. 1d and Supplementary Movie 1, and represent 2- and 3-dimensional (D) renderings, respectively. The epipial cells are densely interwoven and form a matted meshwork that forms a distinct tunic (i.e., the tunica adventitia) that envelopes the primary afferent cerebral vessels (Fig. 1e–g). This layer is adherent to abluminal vessel walls, being distinct from the tunica media (Fig. 1f, g). The epipia is observed to tether arteries to collagen connective tissue elements within the subarachnoid space (SAS) (Fig. 1e–i).

### Partial epipial detachment forms epipial spaces (EPS) within the adventitia of small-to-medium-sized leptomeningeal arteries

**Fig. 2:**
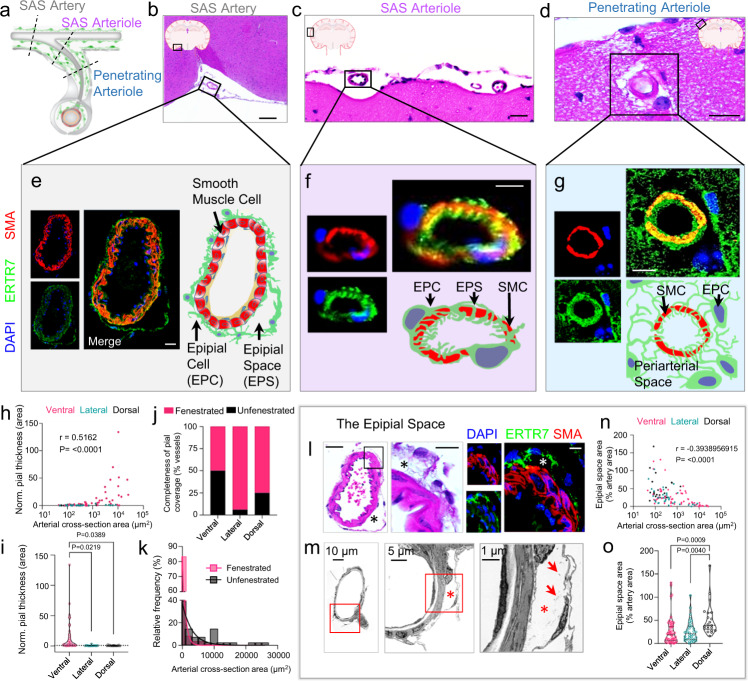
The epipia attenuates and loosens around small-to-medium leptomeningeal arteries, forming the *epipial space*. Schematic (**a**) and routine sections (**b**–**d**) demonstrate the relationships of pial cells on leptomeningeal, i.e., subarachnoid space (SAS) arteries and penetrating arterioles. Medium-sized SAS arteries at the brain surface demonstrate attenuation and loosening of the epipia layer, creating an epipial space (**e**), whereas smaller SAS arteries and penetrating arterioles demonstrate further thinning and coalescence of pial layers with the vessel walls, with occasional envelopment of the arteriolar smooth muscle cell layer (**f**, **g**). Analysis of epipial sheath thickness, expressed as a percentage (%) of vessel area, is shown according to vessel size (**h**) and brain region (**i**); Pearson correlation coefficient; *n* = 86 vessels. Likewise, analysis of epipial fenestration, expressed as a percentage (%) of total vessels, is shown according to brain region (**j**) and vessel size (**k**); one-way ANOVA with Tukey’s multiple comparisons test; *n* = 122 vessels. A medium-sized artery in ventral mouse brain (**l**, the same vessel shown in **e**), is depicted at higher magnification and demonstrates the loosened epipial sleeve composed of interlinking pial cells that partially enclose the epipial space (asterisks). Features of the epipial space are highlighted by immunohistochemistry (**l**, right-hand side), and are shown to advantage in ultrastructural images (**m**). Enlargements of boxed micrograph areas demonstrate the intra-adventitial space, in which scattered collagen fibrils are appreciated (arrows). Analysis of epipial space areas are shown relative to vessel size (**n**); Pearson correlation coefficient; *n* = 115 vessels from 6 mice. Analysis of epipial space areas are shown relative to brain region (**o**); one-way ANOVA with Tukey’s multiple comparisons test; *n* = 115 vessels from 6 mice. (**b**–**d**, L left) H&E; (**e**–**g**, L right) red/CY3, SMA; green/FITC, ERTR7; blue, DAPI; **m** transmission electron micrographs, with scale bars as indicated (asterisks represent the epipial space); scale bars = (**b**) 100 μm; **c**–**e** 20 μm; **f**, **g** 10 μm. Source data are provided as a Source Data file. TEM data are representative of 50 vessels from 3 mice.

We further analyzed vessels of different size within the SAS and discovered that as cerebral arteries and their branches disperse from ventral brain regions and their vessel calibers diminish, the completeness of the epipial layer concomitantly reduces (Fig. 1f, h–m). The epipial cells also gradually loosen and partially detach from abluminal surfaces of small-to-medium-sized leptomeningeal arteries, giving rise to shaggy adventitia (Supplementary Fig. 3a, b) as well as intra-adventitial microcompartments (Fig. 2). Artifactual separation from the vessel walls is excluded, as the spaces are consistently observed on cryo, paraffin and vibratome prepared tissue sections, being reproduced on routine (Fig. 2a–d, l) and immunohistochemistry staining (Fig. 2a, e–g, l) as well as on electron micrographs (Fig. 2m; Supplementary Fig. 3c, d). Given its consistent situation next to the epipial layer, we refer to this cavity as the *epipial space* (EPS). Notably, the EPS is present only at certain regions of the afferent brain vasculature, being most prominent around small vessels at dorsal brain regions (Fig. 2n, o). Loosely packed fibrils of collagen are observable within the spaces (Fig. 2m; Supplementary 3c, d). As afferent vessels approach the brain surface, the epipia attenuates and frequently inserts onto or within the tunica media (Fig. 2c, d, f, g). In a subset of leptomeningeal and/or penetrating arterioles, the epipial processes permeate vessel walls to envelope the vascular smooth muscle cell (SMC) layer before abruptly disappearing (Fig. 2f, g; Supplementary Fig. 3b). On other vessels, the epipia thins and terminates at abluminal vessel margins but remains unattached to the tunica media. As the epipia attenuates, the EPS narrows and becomes slit-like before the sheath and space both disappear (Fig. 2n). The sites of epipial and EPS merging and/or obliteration are variably situated within intracortical or pericortical regions (Fig. 2c, d, f, g).

### Epipia and EPS are only present on a subset of cerebral arteries and arterioles

Small arteries and arterioles converge at cortical brain surfaces with variable epipial coverage (full/partial/none) as summarized according to brain region (ventral, 54%/29%/16%; lateral, 26%/66%/8%; and dorsal, 29%/57%/14%) in Fig. 1k and according to cross-sectional area (0–9999 µm, 37%/48%/15%; 10,000–19,999 µm, 100%/0%/0%; >20,000 µm, 100%/0%/0%) in Fig. 1i, m. When present, epipial sheaths are variably thinned (ventral, 13.19 ± 24.06 µm; lateral, 0.6310 ± 0.8037 µm; dorsal, 0.5611 ± 0.6440 µm; Fig. 2h, i). Before complete loss of coverage, the epipia on thinly sheathed vessels acquire apertures between sparsely packed pial cell processes (Fig. 2j, k). Therefore, as with partially sheathed vessels, the epipial coverings on thinly sheathed vessels are frankly fenestrated (Fig. 2g), with 75% of vessels <10,000 µm in cross-sectional area exhibiting microfenestrae on confocal imaging (Fig. 2k). A summary of fenestration status on afferent vessels according to brain region (ventral, 50%; lateral, 94%; dorsal, 75%) is shown in Fig. 2i. Other regional differences in pial ensheathment pattern are noted: The epipia is significantly thicker at ventral brain regions (i.e., 13.19 ± 24.06 µm, versus 0.6310 ± 0.8037 µm in lateral brain and 0.5611 ± 0.6440 µm in dorsal brain; *p* < 0.05; Fig. 2j), while epipial fenestrae are more frequently observed on vessels in lateral and dorsal brain regions (Figs. 1k; 2i). Large arteries uniformly exhibit thick, complete epipial coverings; however, small vessels are thinly and/or incompletely sheathed (Figs. 1l, 2h); *r* *=* 0.5162, *P* < 0.0001). As the caliber of vessels diminishes in the SAS, relative EPS dimensions also increase (Fig. 1n; *r* = −0.3939, *P* < 0.0001), being most prominent at dorsal brain regions (Fig. 1o; 52.43 ± 35.29% versus 25.43 ± 29.05% in ventral, *P* = 0.0009, and 26.38 ± 22.97% in lateral brain regions; *P*= 0.0040). Confocal images of fully sheathed and partially sheathed leptomeningeal arteries are depicted in Supplementary Movie 2 and Supplementary Movie 3, respectively.

### Periarteriolar spaces (PAS) are present between the intimal pia (or glia limitans) and epipia (or tunica media) of penetrating arterioles and exist in three configurations in young mice

**Fig. 3:**
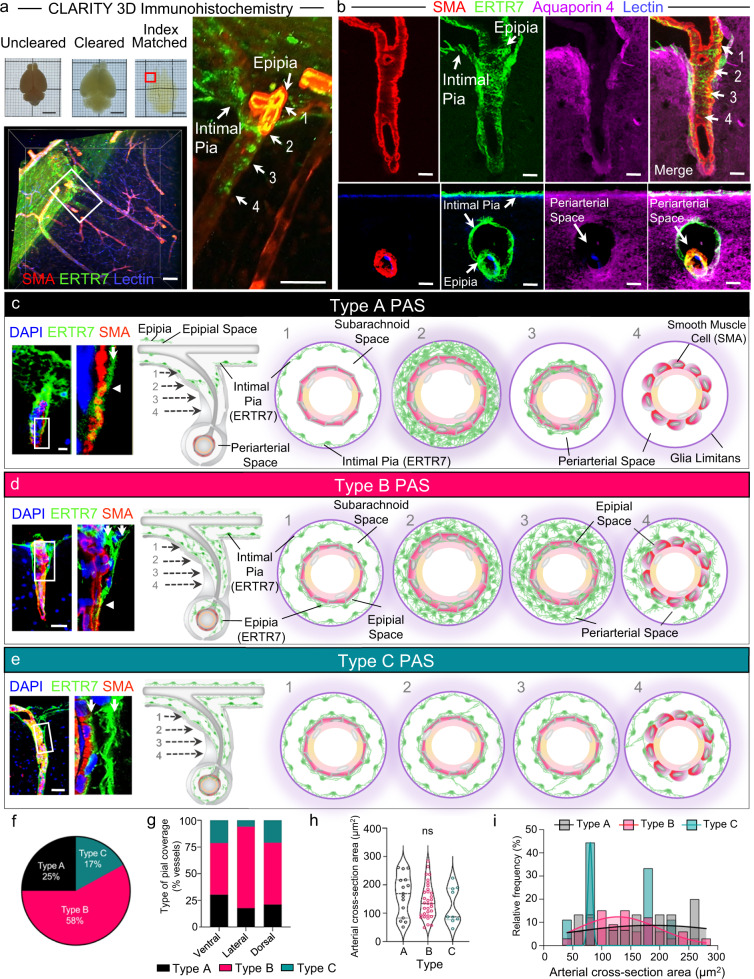
Variability in intimal pial and epipial relationships give rise to distinct PAS architectures in superficial brain regions. As shown on volumetric lightsheet image of a tissue-cleared (CLARITY) specimen, the intimal pia and epipia adjoin at the brain surface and create a basket-like sieve around penetrating arterioles (**a**). The anatomy is further delineated on confocal Z-stack images of longitudinal and axial cortical mouse brain sections (**b**). Heterogeneous relationships of the intimal pia and epipia around penetrating arterioles result in a spectrum of periarteriolar space (PAS) anatomy, with three primary structures in healthy young mouse brains: Type A (**c**), Type B (**d**), or Type C (**e**); white arrows represent separate pial sheaths (i.e., the intimal pia and epipia), while arrowheads mark sites of pial coalescence with the tunica media. The variable anatomy are depicted in longitudinal images with enlargements of boxed areas (**c**–**e**, left-hand side) and axial schematics (**c**–**e**, right-hand side) that depict deeper levels, from left to right (labeled 1–4, respectively). Type A PAS is shown in relation to an unsheathed arteriole (i.e., an arteriole lacking epipial coverage at the site of brain penetration). The distribution of PAS types at the brain surface is summarized in (**f**). PAS types are further characterized according to brain region (**g**) and vessel size (**h** and **i**); one-way ANOVA with Tukey’s multiple comparisons test; *n* = 57 vessels from 6 mice. **a**–**e** Red/CY3, SMA; green/FITC, ERTR7; blue, DAPI or lectin; violet/CY5, aquaporin 4; scale bars = **a**, **e** 50 μm; **b**, **c** 10 μm; **d** 30 μm. Source data are provided as a Source Data file.

In addition to lining brain surfaces and afferent leptomeningeal vessels, the intimal pia also invaginates next to penetrating arterioles (Supplementary Fig. 4), thereby lining the perimeters of intracortical microcompartments. Here, the pial invaginations form shallow, net-like sheaths (i.e. sieves) (Fig. 3a; Supplementary Figs. 4 and 5). As these intracortical spaces in superficial brain regions are distinct from the SAS and EPS, they constitute separate *periarteriolar space* (PAS) regions. Within the PAS, arterioles are often tethered to one side due to asymmetry of secondary penetrating arteriolar branches (Supplementary Fig. 5). Although the superficial PAS compartment is associated with pia, its deep aspect is devoid of any pial lining(s) (Fig. 3a, Supplementary Fig. 5) and the depths of pial penetration are variable. Therefore, the deep aspects of PAS consist of an acellular space (i.e., a thin fissure) that directly borders the glia limitans and arteriolar tunica media. The general relationships of this space with the brain parenchyma (i.e., glia limitans) and penetrating arteriole smooth muscle cell layer (SMC) is appreciated in three dimensional (3D) volumetric images (Fig. 3a, b). A 3D rendering showing net-like pial funnels around penetrating arterioles within a cleared mouse brain specimen is also shown in Supplementary Movie 4.

High-resolution, optical Z-stacked (3D) confocal images demonstrate that the epipial and/or intimal pial components form a perforated funnel around penetrating arterioles (Fig. 3b). Multiplanar imaging of these funnel structures illustrate variations in epipial and intimal pial coverage styles that lead to distinct PAS anatomy. Three generalized patterns of pial anatomy can be found at sites of arteriolar penetration: Type A PAS (25%) are affiliated with single or dual pial lining(s) that merge into a continuous, sheet-like pial reflection that coalesces directly with the arteriolar tunica media (Fig. 3c, f); Type B PAS (58%) are associated with dual pial sheaths (i.e., intimal pia and epipia layers) that interweave to create an alveolated reticulum in the PAS that coalesces with the penetrating arteriolar tunica media (Fig. 3d, f); and Type C PAS (17%) are associated with dual pial sheaths (i.e., intimal pia and epipia layers) that separately line the outer and inner PAS borders, respectively, and remain distinct (i.e., unmerged) at sites of pial termination (Fig. 3e, f). Although Type A and Type B PAS exhibit variably dense, sheet-like pial covers (simple flap or sponge-like, respectively) at areas of PAS–SAS communication, they are finely perforated. In contrast, the dual pial sheaths associated with Type C PAS may contain cell processes but lack intervening pial cell nuclei, thus remaining open and/or uncovered. Retiform pia associated with each PAS type form a variably complex abluminal tissue that tethers intracerebral arterioles as they are received. The distribution of PAS types at the brain surface do not correlate with corresponding brain regions (ventral, 30.3%/48.5%/21.2%; lateral, 17.6%/76.5%/5.9%, dorsal, 21/58/21%) nor with cross-sectional areas of contained vessels (Fig. 3g, h). PAS types are summarized according to vessel size (i.e., cross-sectional areas: Type A, 164.5 ± 72.54 µm^2^; Type B, 142.7 ± 60.64 µm^2^; Type C, 127.3 ± 65.09 µm^2^) in Fig. 3h, i.

### CSF tracer accumulates within PAS and its fate is influenced by intimal pial and epipial anatomy

**Fig. 4:**
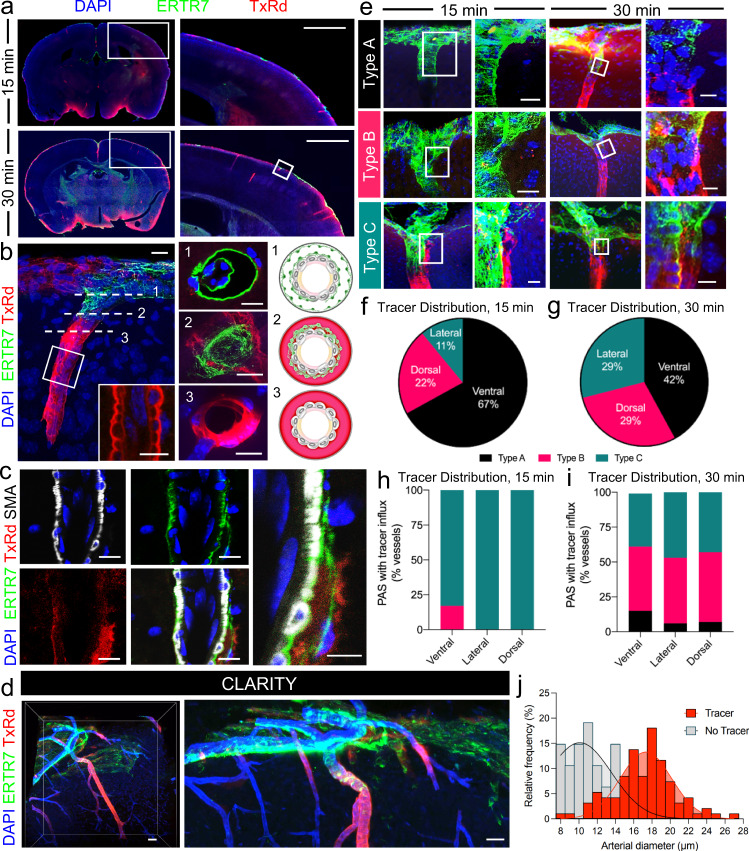
Influx of CSF tracer is delayed, but not restricted across “covered” Type A and Type B PAS. Distribution of an immunofluorescent tracer (Texas Red conjugated to bovine serum albumin, TxRd; mw 66 kDa) was evaluated in mice sacrificed at 15 versus 30 min following slow intracisternal infusion (**a**), and revealed heterogeneous spatiotemporal deposition patterns as shown in right-hand images that represent the boxed areas. Enlargement of the small box at lower right panel in **a** illustrates tracer passage into a Type B PAS (**b**). Enlarged inset depicts pooling of tracer in PAS, creating a scalloped appearance around smooth muscle cells (**b**, inset). Cross-sectional images from axial sections (labeled 1–3) of another Type B PAS is shown in the middle panel and the anatomic relationships are summarized in axial schematics on right-hand side. Triple labeling of a Type B penetrating arteriole (shown in longitudinal section) depicts TxRd tracer signal around the ERTR7-labeled pial cell elements (**c**). Volumetric lightsheet image of a tissue-cleared (CLARITY) specimen further demonstrates variable penetration of the tracer (**d**). Examples of tracer-positive PAS are shown (**e**) and distributions are quantified in different brain regions at 15 min (**f**, **h**) and 30 min (**g**, **i**) post-infusion; *n* = 57 vessels from 6 mice. **j** At 30 min post-infusion, tracer was mostly found around large diameter arteries; *n* = 141 vessels from 6 mice. Red/Texas Red, TxRd; green/FITC, ERTR7; white/CY5, SMA; blue, DAPI or lectin; scale bars = **a** 500 μm; **b**–**e** 10 μm. Source data are provided as a Source Data file.

To discern the directionality and mechanism of CSF movement at the brain surface, we next studied patterns of bovine serum albumin-Texas Red (66 kDa; TxRd) tracer deposition following slow infusion into cisterna magna of live young mice. Free fluorescent tracer was observed to readily traverse the pial layers. On epifluorescence and confocal imaging, TxRd was detectable within PAS but not EPS (Fig. 4a–e). However, the efficiency of free tracer accumulation into deep PAS regions is found to be variable (Fig. 4e), as time-dependent tracer measurements of fluorescence intensity appeared to be influenced by PAS anatomy (Fig. 4e). The distribution of tracer positive PAS at 15 min post-injection was primarily restricted to the ventral cortex (67%) compared to the lateral and dorsal cortices (11% and 22%, respectively) (Type A/B/C: ventral, 0%/17%/83%; lateral, 0%/0%/100%; dorsal, 0%/0%/100%), as summarized in Fig. 4e, f, h. Likewise, the distribution of tracer positive PAS anatomy at 30 min post-injection (Type A/B/C: ventral, 15%/46%/38%; lateral, 6%/47%/47%; dorsal, 7%/50%/43%) is summarized in Fig. 4e, g, i. At 30 min post-infusion, type A PAS frequently exhibited superficial trapping of the tracer bolus (Fig. 4e). Precipitated tracer material was also observed in superficial aspects of type A and B but not type C PAS. The distribution of tracer-positive versus tracer-negative arterioles 30 min post-infusion is illustrated in Fig. 4d and quantified in Fig. 4j, and show preferential tracer accumulation alongside larger caliber vessels. Volumetric (3D) rendering of a penetrating arteriole with adjacent PAS tracer from a cleared mouse brain is shown in Supplementary Movie 5. Additional videos depicting types A–C PAS with accumulated tracer at 30 min post-infusion are shown in Supplementary Movies 6–8, respectively.

### Pial layers do not present a barrier to CSF flow in vivo

**Fig. 5:**
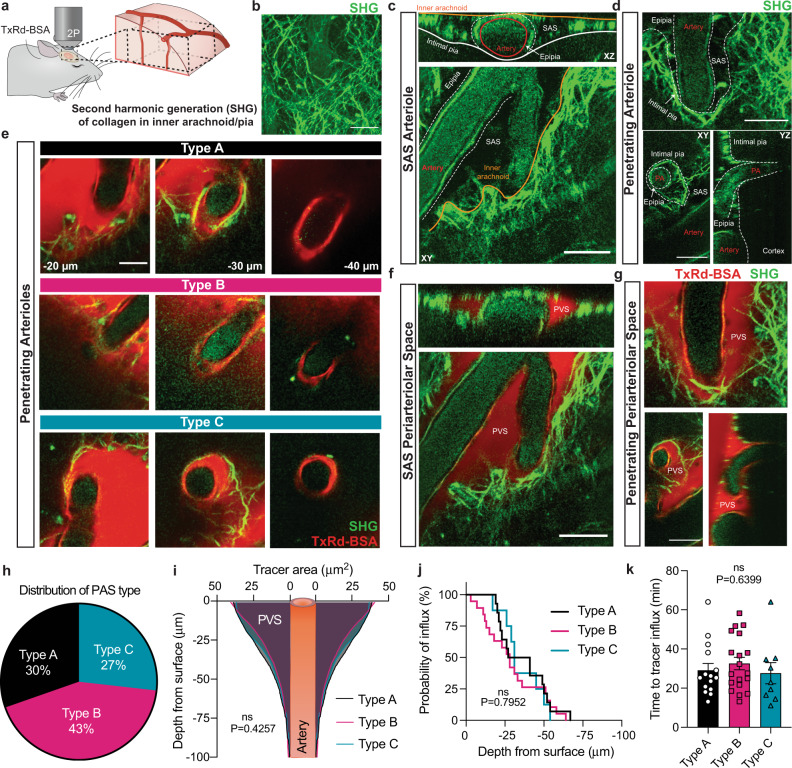
The pia is not a barrier to CSF tracer transport (size 66 kDa), irrespective of periarteriolar space (PAS) type. **a** A CSF tracer (bovine serum albumin conjugated to Texas Red, TxRd-BSA; 66 kDa) was injected into the cisterna magna of live ketamine–xylazine anesthetized mice. After dura had been removed, the pial surface and CSF tracer movement patterns were imaged through a cranial window using two-photon (2P) laser scanning microscopy. **b** Second harmonic generation (SHG) was used to visualize the collagen fibers in the inner arachnoid and pia. **c** SHG imaging of an arteriole in the subarachnoid space (SAS), with an orthogonal reconstruction in the *XZ* plane (top panel), showing the inner arachnoid overlying the vessel surrounded by epipia coursing within the SAS on top the intimal pial layer. **d** SHG imaging of a penetrating arteriole (PA) showing how the epipia coalesces with the intimal pia as it dives down into cerebral cortex. Bottom right: *YZ* orthogonal view of the PA entering cortex. **e** Representative images of each PAS type surrounding penetrating arterioles at −20, −30, −40 µm below the cortical surface: Type A (top panel), Type B (middle panel), Type C (bottom panel). **f** CSF tracer in the surface perivascular spaces (PVS) is surrounded by the inner arachnoid superiorly, the intimal pia inferiorly, the fusion of the inner arachnoid/intimal pia laterally, and the epipia of the accompanying artery medially. This space coincides with the SAS of the surrounding leptomeningeal arterioles. **g** CSF tracer enters penetrating PAS and traverses across the merged epipial and intimal pial membranes. **h** Distribution of PAS by type (*n* = 42 PVS from 8 mice). **i** Area (µm^2^) of tracer coverage in the PVS as a function of depth from the brain surface. One-way ANOVA with Tukey’s post hoc for multiple comparisons, *P* = 0.4257, ns: not significant. **j** Probability of observing CSF tracer influx at varying depths of PAS type at 60 min after injection. Log-rank (Mantel–Cox) test, *P* = 0.7952, ns. **k** Time to tracer influx after the start of the intracisternal tracer injection in minutes (min). One-way ANOVA, *P* = 0.6399, ns. Scale bars = **b**–**d**, **f**, **g** 20 µm. Data are presented as mean ± SEM. Source data are provided as a Source Data file.

The role that intimal pial-epipial merging has on penetrating arterioles and CSF inflow into brain is unknown. Some believe that pia separates two independent flow streams: One that enters the brain between the pia and the glia limitans and another that leaves the brain alongside the vascular basement membrane^14^, while others believe a pial reflection forms a blind ended pouch that limits CSF entry to brain^17^. To test the role that pial layers play on CSF transport in vivo we next sought out to use two-photon laser scanning microscopy to analyze real time flow routes and kinetics of CSF influx into PVS in live anesthetized animals. However, ERTR7 is an intracellular antigen that preferentially labels cells rather than the constituents of the pial extracellular matrix; we therefore asked whether collagen could be employed as a surrogate to visualize pial structure in vivo^21^? To test this hypothesis, we evaluated the co-localization of ERTR7 label and a pan-collagen label (Supplementary Fig. 6a, b) and found that ERTR7 exhibited a high degree of co-localization with collagen independent of the PAS type (Supplementary Fig. 6c, Type A: 88.07%, Type B: 90.30%, Type C: 92.01%; *P* = 0.0566). We next evaluated if a blinded rater would be able to identify PAS types using either an ERTR7 or collagen label and found a high degree of intra-rater agreement (Supplementary Fig. 6d, Type A: 100%, Type B: 96.7%, Type C: 93.3%), supporting that collagen is an adequate marker of pia and PAS type. To image pia in vivo, we employed second harmonic generation (SHG) to visualize collagen fibers in the brain of live mice after intracisternal tracer delivery (TxRd, Fig. 5a, b). SHG of the pial surface identified PVS around arterioles in the subarachnoid space (Fig. 5c) and the penetrating arterioles (Fig. 5d). Using this method, type A, type B, and type C PVS were visualized at cortical brain surfaces of live mice following intracisternal injection of fluorescent TxRd-BSA (Fig. 5e). Tracer was first observed entering the brain along surface PVS that accompany the arterioles within the subarachnoid space (Fig. 5f) and underwent transpial movement to influx into deep PVS regions alongside penetrating arterioles (Fig. 5g). Analysis of all penetrating arterioles (*n* = 43 from 8 animals) showed a similar distribution of PAS types A–C (Fig. 5h, Type A/B/C: 30%/43%/27%) as seen prior on ex vivo analyses (Fig. 3f). CSF tracer was readily observed to cross pial fibers to enter type A (Supplementary Movie 9), type B (Supplementary Movie 10), and type C PVS (Fig. 5e, Supplementary Movie 11). PVS size as labeled by the area covered by the CSF tracer did not differ between PAS type (Fig. 5i, *P* = 0.4257) and the probability of tracer influx at 60 min after injection was also similar between all three PAS types (Fig. 5j, *P* = 0.7952). CSF flow into penetrating PVS was observed ~30 min after intracisternal injection (Fig. 5k, Type A: 29.1 ± 3.5 min; Type B: 32.6 ± 3.2 min; Type C: 27.6 ± 5.4 min, *P* = 0.6399), confirming continuity of the PVS with the subarachnoid space. Overall, data obtained from live mice confirm that despite differential pial anatomy in brain PVS, pia does not present an impermeable barrier to small molecules and/or CSF flow.

### Pial anatomy and PAS architecture are altered in aged and APP/PS1 mice

**Fig. 6:**
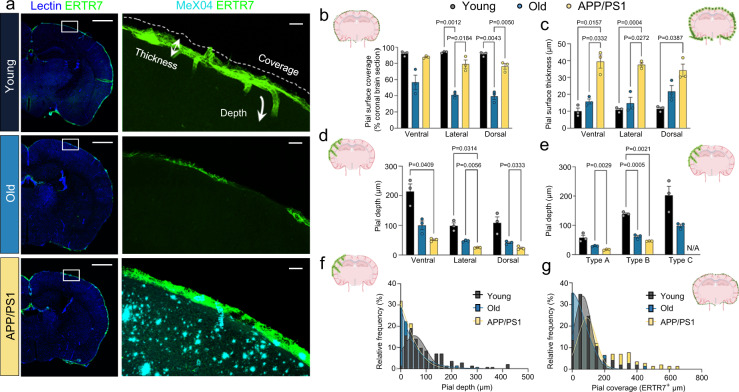
Pial coverage, thickness and depth of penetration vary with age, brain region, and PAS type in young, old, and APP/PS1 mice. ERTR7-labeled coronal brain sections from young (2 months), old (13 months), and APP/PS1 (13 months) mice demonstrate pial coverage at the brain surface (**a**). Notice variable coverage, thickness, and depths of penetration. Analyses of percent (%) intimal pial/surface coverage (**b**), intimal pial/surface thickness (µm) (**c**), and depth of pial penetration (µm) according to brain region (**d**), and PAS type (**e**) are shown; two-way ANOVA with Tukey’s multiple comparisons test. The overall distribution of pial depths and areas (µm^2^) of pial coverage surrounding penetrating arterioles are shown in plots **f** and **g**, respectively. Young: *n* = 141 vessels from 3 mice; Old: *n* = 139 vessels from 3 mice; APP/PS1: *n* = 141 vessels from 3 mice. Green/FITC, ERTR7; cyan/CY5, MeX04; blue, lectin; scale bars = **a**–**c**, left 500 μm; **a**–**c**, right 50 μm. Data are presented as mean ± SEM. Source data are provided as a Source Data file.

**Fig. 7:**
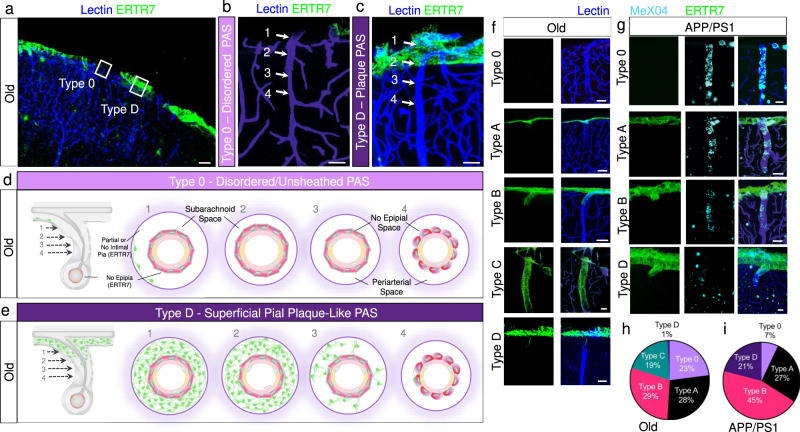
Disordered and plaque-like PAS appear with aging along with conversion to covered or “closed” PAS types in APP/PS1 mice. In old (13 months) mice, pial distribution is irregular at the brain surface and around penetrating arterioles (**a**), resulting in PAS without any pial coverage, i.e., Type 0 (**b**), and PAS with thickened, superficial plaque-like coverage, i.e., Type D (**c**), in addition to usual type PAS (i.e., Type **a**–**c**). The anatomy of Type 0 and Type D PAS are depicted in schematics shown in (**d**) and (**e**), respectively. PAS types observed in old and APP/PS1 mice are illustrated in (**f**) and (**g**). The overall distribution of PAS types at the cerebral cortical brain surface of old mice is summarized in (**h**). The overall distribution of PAS types at the cerebral cortical brain surface of APP/PS1 mice is summarized in (**i**). Note diminishment of type 0 and absence of type C PAS in APP/PS1 mice. Old: *n* = 139 vessels from 3 mice; APP/PS1: *n* = 141 vessels from 3 mice. Cyan/CY5, MeX04; blue, lectin; green/FITC, ERTR7. Scale bars = **a** 50 μm; **b**, **c** 10 μm; **f**, **g** 20 µm.

To elucidate the depths of pial penetration within cerebral cortices as well as any structural changes of pia mater that may occur with aging, ERTR7-labeled coronal brain sections were next compared between young (2-month-old) wildtype (WT) mice, old (13-month-old) WT mice, and old APP/PS1 mice (13-month-old AD model). These experiments demonstrated significant differences in pial anatomy among the three groups (Fig. 6a). Mean percentage of intimal pial coverage varied for young (92.48 ± 0.73%), old (45.67 ± 5.52%), and APP/PS1 mice (81.07 ± 3.45%; Fig. 6b). Likewise, the intimal pial thickness was 10.78 ± 0.42 µm for young, 17.44 ± 2.19 µm for old WT, and 37.07 ± 1.527 for old APP/PS1 mice (Fig. 6c). Among ventral/lateral/dorsal brain regions, the average depth of pial penetration was 140 ± 37 µm for young, 63 ± 19 µm for old WT, and 33 ± 9 µm for old APP/PS1 mice (Fig. 6d) and this varied depending on the type of PAS (Fig. 6e). Type C spaces had the deepest pial coverage (203 ± 31 µm), followed by type B (139 ± 4 µm) and type A spaces (58 ± 8 µm) (Fig. 6e). Pial penetration was shallower among old animals relative to young animals, but shared a similar overall pattern with pia associated with type C PAS penetrating most deeply (99 ± 6 µm) followed by type B (61 ± 4 µm) and type A (29 ± 1 µm). Compared to age-matched controls, APP/PS1 mice exhibited shallower pial penetration among type A PAS (17 ± 1 µm) and type B PAS (46.1 ± 0.3 µm), yet type C spaces were notably absent (Figs. 6e, 7). Plots showing the distributions of pial depths (i.e., ERTR7 penetration) and pial coverage (i.e., total ERTR7 pixel areas) around penetrating arterioles are depicted in Fig. 6f, g, respectively, and demonstrate a wide spread of values in young WT mice, with notable blunting in old WT animals. Immunofluorescence labeling demonstrate the irregularity of the intimal pia layer in the old animals (Fig. 7a) with patchy loss of pia (Fig. 7a, b) and patchy expansion of pia (Fig. 7a, c) seen in adjacent areas. The unevenness of ERTR7 distribution manifested through presence of unsheathed (Type 0) PAS (Fig. 7b, d) and plaque-type (Type D) PAS (Fig. 7c, d) in old WT animals that were not observed in the young WT mice. The morphology of type D PAS in old mice suggests that it may represent a modified type A/B PAS variety that underwent pial expansion. Overall, the analyses showed shorter, thicker PAS coverage styles in APP/PS1 mice relative to WT mice that were appreciated among vessels both with and without amyloid, as assessed by MeX04 label. Compared to old WT mice, old APP/PS1 mice demonstrate significant difference in PAS distribution (*P* < 0.0001) with apparent conversion to covered/closed PAS types (i.e., presence of types A,B,D versus types 0 or C; Fig. 7f–i). In old APP/PS1 mice, amyloid was observed in EPS regions, as shown in an orthogonal confocal image (Supplementary Fig. 7), and was seen in all PAS types (Fig. 7e, g; Supplementary Figs. 8–11) including in peri-arterial and peri-arteriolar areas that lacked any significant pial coverage (Fig. 7f–h; Supplementary Figs. 10 and 12). Confocal optical Z-stack images of penetrating arterioles from an APP/PS1 mouse with presence of MeX04 and TxRd tracer label are shown in Supplementary Movies 12 and  13.

### CSF tracer deposition patterns are variable in young, old, and APP/PS1 mice and are associated with variable PAS macrophagic response

**Fig. 8:**
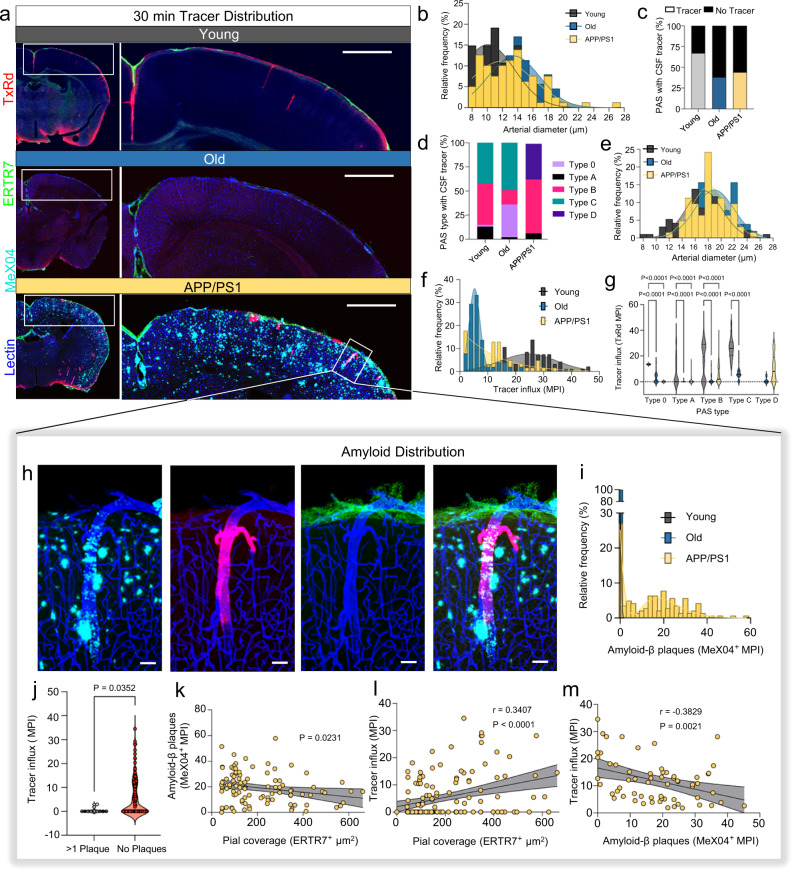
CSF tracer accumulation patterns are variable within PAS of healthy, old, and APP/PS1 mice, and correlate with perivascular MeX04 (i.e., amyloid-β plaque) and ERTR7 (i.e., pial cell) density. Coronal images demonstrate TxRd tracer accumulation in brains of young, old and APP/PS1 mice (**a**). The distribution of vessel sizes in young, old and APP/PS1 mice are shown (**b**) along with the proportion of tracer-positive PAS (**c**) and tracer positivity according to PAS type (**d**). Distribution of tracer-positive PAS are further depicted in the three groups according to arterial diameter (**e**). The overall distribution of tracer mean pixel intensity (MPI) is shown (**f**) along with distribution of MPI according to PAS type (**g**); two-way ANOVA with Tukey’s multiple comparisons test. A penetrating arteriole from APP/PS1 mouse is shown at higher power (**h**) and illustrates diminishment of tracer accumulation below PAS abutting plaques, with brisk fluorescence in secondary penetrating arterioles, possibly representative of regurgitant flow. The distribution of MeX04 (amyloid-β plaque) label according to MPI is summarized in (**i**). TxRd tracer accumulation (MPI) is summarized in plaque-positive (>1 PAS abutting plaques) versus plaque-negative PAS in (**j**), showing marked diminishment of tracer accumulation that may be indicative of decreased flow; unpaired *t*-test. Correlation of MeX04 with ERTR7 pixel are is shown in (**k**); simple linear regression with 95% CI. Correlation of tracer MPI with ERTR7 label is shown in (**l**); simple linear regression with 95% CI. Correlation of tracer MPI with MeX04 MPI is shown in (**m**); simple linear regression with 95% CI. Cyan/CY5, MeX04; blue, lectin; green/FITC, ERTR7; red/Texas Red, TxRd. Scale bars = (**a**) 500 μm; (**h**) 50 μm. Data are depicted 30 min following intracisternal tracer infusion. Young: *n* = 141 vessels from 3 mice; Old: *n* = 139 vessels from 3 mice; APP/PS1: *n* = 141 vessels from 3 mice. Source data are provided as a Source Data file.

**Fig. 9:**
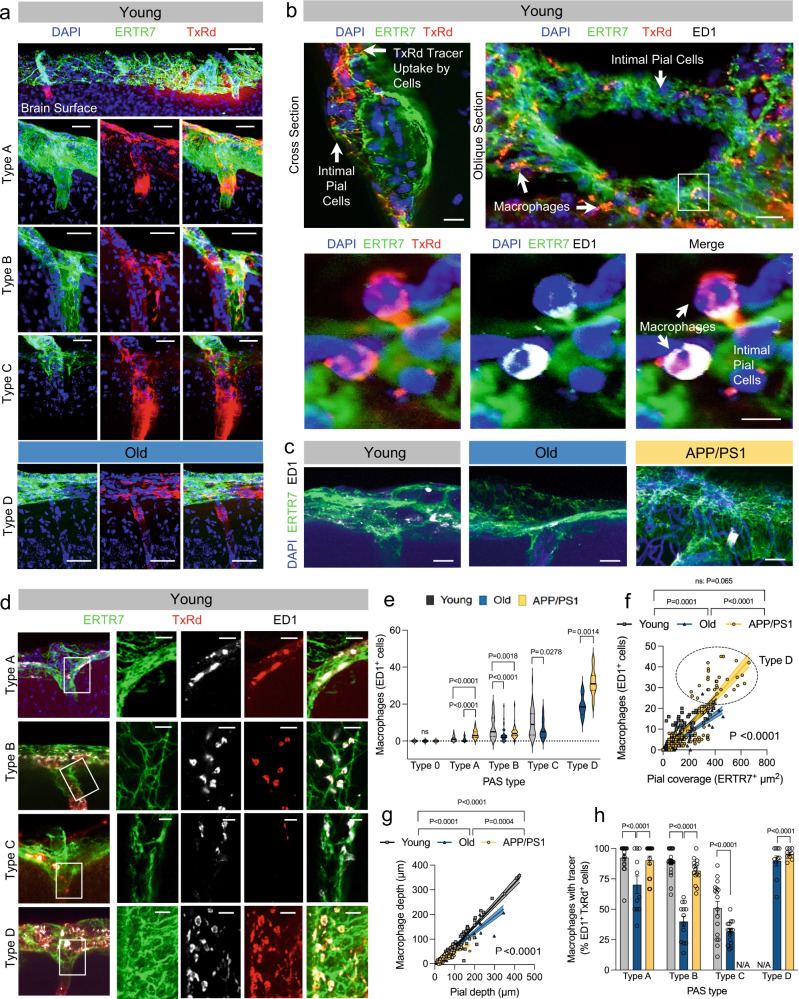
CSF tracer undergoes differential macrophagic processing within distinct PAS types. At sites of arteriolar penetration (**a**), the intimal pia and epipia merge and distinct patterns of tracer deposition are observed around ensheathed vessels (i.e., Type A-D PAS), with free tracer passage, stagnation of the tracer bolus (open arrow) and/or cellular sequestration of tracer (solid arrows) noted. Cross and oblique PAS sections (**b**, lower panel) depict tracer-positive cells studding the ERTR7-positive PAS cell network. As demonstrated in the oblique section and lower panel representing an enlargement of the boxed area, the TxRd tracer positive cells represent ED1-positive macrophages. ED1-positive cells are shown within Type B PAS of young, old, and APP/PS1 mice (**c**) and the distribution of tracer among ED1-positive macrophages in type A–D PAS of young mice is shown in (**d**). The number of ED1-expressing cells is variable among PAS types, being prominent in type D PAS (**e**) and correlating strongly with ERTR7 density (**f**); *n* = 107 vessels. Two-way ANOVA with Tukey’s multiple comparisons test in D. Simple linear regression with 95% CI in E, F; *P* values in legend refer to testing between slopes. Moreover, the depths of ED1-positive macrophages in PAS correlate strongly with pial (i.e., ERTR7) depth (**g**). The distribution of tracer among ED1-positive macrophages in type A–D PAS is quantified in (**h**). Two-way ANOVA with Tukey’s multiple comparisons test in (**h**). **a**–**g** Red/Texas Red, TxRd; green/FITC, ERTR7; white/CY5, ED1; blue, DAPI; Scale bars = (**a**, upper panel) 30 μm; (**a**, lower panels, **b** upper panel, **c**, **g**) 10 μm; (**b**, lower panel) 5 µm. Young: *n* = 87 vessels from 3 mice; Old: *n* = 71 vessels from 3 mice; APP/PS1: *n* = 66 vessels from 3 mice. Data are presented as mean ± SEM. Source data are provided as a Source Data file.

In evaluating 30-min ex vivo tracer deposition patterns, marked differences in TxRd accumulation are observed in brains of young, old and APP/PS1 mice (Fig. 8a–g). The distribution of tracer-positive PAS in young, old, and APP/PS1 mice are shown in Fig. 8d and are further characterized according to vessel size in Fig. 8e. While spread of tracer according to vessel size is similar among the groups, its mean pixel intensity (MPI) is markedly reduced in old WT mice (Fig. 8f), being most prominent in type C PAS (Fig. 8g). In contrast, increased MPI of TxRd tracer is observed in APP/PS1 mice along vessels of variable size. While plaque load around vessels is found to be variable in APP/PS1 mice (Fig. 8h, i), significant diminishment of tracer signal is noted in PAS that exhibit > 1 abutting plaques (i.e., 2 or more) compared to PAS without any plaques (*P* < 0.05) (Fig. 8h, j). ERTR7 signal correlated inversely with amyloid deposition, as assessed by MPI of MeX04 signal within individual PAS (*P* < 0.05) (Fig. 8k). While tracer influx was not found to be inhibited by ERTR7-positive pial ensheathments (Fig. 8l), it correlated inversely with the degree of amyloid deposits (Fig. 8m). On high-power imaging, cellular sequestration of tracer is also noted within PAS (Fig. 9a). Images of PAS cross section (Fig. 9b, left panel) and oblique section (Fig. 9b, right and lower panels) depict the accumulation of TxRd tracer within PAS cells. Oblique (Fig. 9b, right and lower panels) and coronal (Fig. 9c) sections further demonstrate the presence of ED1-positive macrophages within PAS. Co-localization of TxRd tracer within these ED1-positive cells that localize to ERTR7-positive PAS tissue is shown in Fig. 9b (right and lower panels) and Fig. 9d. The relative number of ED1-expressing cells is found to be variable among distinct PAS types, being prominent in Type D PAS that are present in old mice (Fig. 9e) and strongly correlating with ERTR7 signal density (Fig. 9f). Depths of macrophage penetration within PAS also correlate strongly with depths of PAS pial penetration (Fig. 9g). Moreover, the relative proportion of macrophages exhibiting evidence of tracer within PAS varied with PAS type, being most prominent in closed PAS types (i.e., types A, B, and D) (Fig. 9h).

## Discussion

This study, which is the first to systematically analyze pial morphology across cerebral cortical PVS in mice, reports the boundaries of anatomic compartments that exist next to afferent cerebral vasculature in mouse brain. Using ERTR7, a recently described pial marker, two PVS compartments are revealed and are formed by juxta-arterial pial reflections: The first space that we term the epipial space (EPS) (referred to as the *perivascular space* in some prior reports^17^) corresponds to a compartment that arises from intra-adventitial fissures within the walls of leptomeningeal vessels, being bound by the epipia. The second space that we term the periarteriolar space (PAS) (called the *paravascular space* in some prior reports^17^) corresponds to apertures that localize to abluminal aspects of penetrating arterioles, being proximally bound by the intimal pia. Histologic features suggest that these spaces represent partially segregated microcompartments that communicate with the SAS and with each another in superficial brain regions. Thereby, these findings provide a critical reappraisal on meningeal anatomy and refine knowledge regarding the microstructure of the meningovascular unit. Moreover, the anatomic features of these spaces and the cells that line them suggest functions of brain pia as a neuroimmune interface that partly compartmentalizes the SAS while scaffolding macrophagic cells within partially segregated PVS compartments.

The histology of ERTR7-expressing pial cells was interrogated in this study using ultrastructural, light microscopic and immunofluorescent techniques. While electron micrographs revealed partial cohesion of pial cells through intercellular tight junctions, 3D reconstructive immunofluorescent imaging clearly demonstrated that the cells are reticulated. Through high-resolution, volumetric and multiplanar imaging, we show that reticulated cytology accounts for the presence of microfenestrae within interconnecting pial sheaths, producing irregular gaps in the dual pial layers. Micrographs further depict close apposition of these cells with collagen fibrils in EPS/PAS and in sub-intimal pial regions, yet electron-dense membranes consistent with pial basal lamina were not identified on cerebral cortical and SAS imaging. Moreover, light microscopic evidence of discrete basement membranes was not seen. Therefore, it is concluded that pial cells form a microperforated, fibroblastic, lace-like reticulum that associates directly with the glia limitans and with SMC in perivascular space regions. These data challenge the long-held historical perspective that cells constituting the pia mater are epithelioid and rest on a basement membrane, thereby forming a fluid impenetrable, membranous layer. Moreover, results of this study demonstrate heterogeneity of fibroblastic pial cell density and coverage styles around penetrating vessels that lead to distinct PAS morphology in young mice: Type A that consist of sheet-like covers; Type B that consist of alveolated reticulae; and Type C that lacked any significant PAS covering. Prior studies aiming to describe the anatomy of PVS in mammalian brain incidentally have arrived at different conclusions: Hannocks describes PVS to be Type A-like in mice^19^ while Pizzo describes Type B-like PVS in rats^12^ and Zhang describes Type C-like PVS in humans^13^. The present study highlights the importance of quantitative, high-resolution, and volumetric imaging techniques for PVS analyses, as it revealed here that its organization in mice is not stereotyped. Rather, an assortment of PAS architectures is discovered, exhibiting variable structure and degree of patency around penetrating vessels that are independent of accompanying vessel caliber and/or brain region.

In the absence of coherent, epithelial pial membranes, the barrier properties of pia mater were next analyzed by administering fluorophore-labelled tracers into cisterna magna of live mice and investigating their ex vivo *d*eposition patterns following variable intervals post-infusion. Confocal images of injected mouse brains reproducibly depicted fluorescent tracer signal within PAS, despite the presence of the periarteriolar pial ensheathments. This discovery is consistent with two-photon imaging data previously reported in live mice^16^. On confocal imaging, accumulated tracer was readily visualized between pial cell processes and was seen in association with all PAS types, consistent with paracellular movement in the PAS cavities. In light of cytologic evidence depicting pial fenestrae as well as histologic absence of any basement membrane and/or pial cell vesicular change, the findings argue against active and/or basement membrane transport. Thus, these data indicate that pial “membranes” do not represent true barriers. While prior reports have alluded to the presence of dual pial linings^6,22^, the cytology and unionization of the dual layers, and their nature of termination along brain vasculature were previously unknown. We show that heterogeneous compartments around penetrating arterioles are formed in mice by irregularly configured, perforated pial linings that variably merge into arteriolar tunica mediae. Interestingly, ex vivo findings suggested that PAS type differentially affected CSF tracer transport; however, in vivo CSF tracer experiments did not reveal differences in tracer transport according to PVS types. This supports the value of evaluating CSF transport in live animals (i.e., in vivo) with time lapse imaging in addition to studying discrete groups of time-varying post-mortem (i.e., ex vivo) specimens. However, differences between in vivo and ex vivo experiments might also be the result of sampling error since two-photon imaging is restricted to the dorsal cortex which only accounts for 22% of PAS sampled at 15 min in the ex vivo group. At 30 min post injection, ex vivo data become more comparable to in vivo data which is in proximity to the time point at which tracer began appearing at dorsal cortex. Intuitively, it also seems reasonable that pial coverage would not present a source of hydraulic resistance to CSF flow, as this would most likely result in generation of abnormal pressure gradients across the delicate pial meshwork. Nonetheless, these anatomic variations appear to have physiological significance as pertains to patterns of CSF tracer uptake (i.e., phagocytosis by PVS macrophages), temporality of PVS macrophage accumulation, and amyloid deposition in PVS. Given these findings, future work is needed to further elucidate the strategic localization of pial macrophages at entry sites of PVS, their roles in innate and adaptive immune signaling, and their roles in brain maintenance including in removal of amyloid from blood vessels in diseases like Alzheimer’s and cerebral amyloid angiopathy (CAA).

Relative to uncovered PAS (type C), covered PAS (i.e., type A/B) exhibited accentuated phagocytic response to accumulated tracer in young mice. At 30 min post injection, ED1-positive macrophages with ingested tracer were readily evident in superficial PAS, being prominent in type A/B but not type C vessels. More notably, a significant positive correlation between ERTR7 expression and ED1-positive cell count was noted within the PAS chambers, suggesting that pial reticulae moderate phagocytic responses and free movement of tracer, which may facilitate innate neuroimmune processing of inflowing fluid. While the pial tissue was permissive of free tracer movement, precipitated tracer and ED1 expressing cells were restricted from entering depths of PAS, suggesting size-dependent flow of CSF content^12,16^. While this data using a 66 kDa cisternal tracer shows that pia does not restrict entry of small molecules, prior work documenting limitations of entry to 1 µm particles^23^ and to scaffolded pial cells seen here suggests a sieve-like function of this tissue and implies a critical role for the pia in CSF processing. The arrangement of perforated pial coverings and ED1 signal around penetrating vessels also suggest that the pia provides an adhesive substratum, similar to ERTR7 expressing cells present within lymph nodes that convey fluid while permitting filtration, scavenger function and the surveillance of lymph^21^. Moreover, the superficial ERTR7-positive pial coverings on type A/B vessels may likely correspond to the so-called VRS^24^ that over the years have been thought to be completely enclosed.

While free low-molecular-weight tracer accumulated substantially within PAS, it was not detected to significant degree within EPS, indicating that CSF permeation (i.e., glymphatic influx) occurs predominantly via PAS conduits. However, tracer-laden macrophages were present within distal EPS regions, indicating functional continuity of this space with the SAS and PAS chambers. In fact, findings of this study suggest that EPS, SAS, and PAS are only partially enclosed (i.e., they are in continuum) at the brain surface and argue against divergent, opposing flow in different directions that was recently hypothesized at the brain surface involving *perivascular* and *paravascular* spaces^14^. Moreover, the presence of tracer-laden macrophages in EPS may likewise indicate that the epipia, similar to the intimal pia, may function vitally to entrap CSF antigen. As the unique cytology and spatial organization of pial cells around afferent cerebral vessels suggests a site of antigen and/or microparticle trapping, the pia may have facilitative roles in phagocyte functions such as in antigen presentation and adaptive immune signaling^25^ and in this manner may have functions similar to ERTR7-expressing stromal cells that exist around high-endothelial venules^21^. ERTR7 stromal elements have also been shown to define microanatomic subcompartments that are dynamic in lymph nodes where they remodel in response to antigenic challenges^25^. In light of this, the functions of the EPS/PAS and the nature of cells contained in them should be further researched.

Notably, this study also demonstrates that significant changes in pial structure occur with aging. In 13-month-old WT mice, the intimal pia was found to be irregular and exhibit patchy loss of coverage involving large regions of cortical brain. Additionally, its thickness varied widely in older animals. Heterogeneity of the intimal pia layer led to PAS without any pia coverage (type 0) as well as adjacent PAS with thickened plaque-like pial ensheathments (type D), in addition to usual style (types A–C) PAS coverage. Moreover, mean depths of pial penetration among old mice were significantly attenuated in different brain regions and PAS types compared to young WT animals. While the reasons for age-related diminishment of pial coverage are unknown, the histologic changes may likely impact on neuroimmune function at superficial brain regions. Indeed, marked diminishment of ED1 cell reaction was noted concomitantly with age-related ERTR7 (pial) fiber attenuation. Interestingly, examination of age-matched APP/PS1 mice revealed thickening of the pia with increased PAS coverage, including conversion to covered and/or plaque-type pial coverage styles that correlated with enhanced ED1 response in amyloid-diseased brain regions. Moreover, the macrophages in covered (especially type D) PAS exhibited enhanced phagocytic response to tracer that may be due to increased access and/or exposure to antigens in these covered PAS types. MeX04-positive amyloid was also identified within the pial layers, involving both the EPS and PAS, further suggesting that the brain pia may critically function in the trapping of CSF antigen.

While the results of this study elucidate PVS anatomy, they raise further questions regarding amyloid processing and metabolism. It has long been known that incipient CAA lesions consist of amyloid deposits within the tunica adventitia and tunica media of small, afferent cortical and leptomeningeal vessels. In combination with the glymphatic hypothesis, our findings into PVS anatomy provide clues into the nature of fluid movement and amyloid deposition at the brain surface. Impairment of glymphatic flow may cause stasis in these perivascular space compartments, as suggested here in APP/PS1 animals in which ERTR7, TxRd tracer, and ED1 showed inverse correlation with MeX04 label. While causality cannot be discerned from this study, amyloid deposits in PAS may cause destruction of pial and/or macrophage elements while inhibiting glymphatic influx, and may thereby impact on neuroimmunity. Alternatively, diminishment of glymphatic flow and attenuation of pial and macrophagic elements in PAS may predispose to amyloid deposition which may subsequently induce a pernicious cycle of glymphatic failure with increasing β-amyloid accumulation. While the roles of amyloid within the brain remain unclear^26^, and its clearance via SMC pathways and drainage into blood plasma are described^27^, the observation of β-amyloid deposits adherent to the abluminal aspects of vessel walls and prominently enmeshed within the pial layers in EPS and PAS, and in the presence of dense macrophages suggest that brain pia may be a separate therapeutic target for neuroimmune β-amyloid processing.

This study offers anatomical evidence and illustrates an intimate association of vascular SMC and the glia limitans with intracranial fluid, as well as with dual pial layers. Thus, it presents a revised model of PVS by which perivascular physiology in the mouse brain may be analyzed. The data may have potential broad significance into processes involved with fluid, cellular, solute, and waste regulation in mammalian brain. However, the limitations of this analysis should be noted. As this study primarily evaluated post-mortem brain tissues derived from mice, expanded investigations are needed to better understand patterns of in vivo fluid flow. In our experiments, the origin(s) of ED1-positive cells were not assessed and may represent resident and/or recruited population(s). Accordingly, future work should investigate precise lineage(s) of phagocytes in EPS/PAS to elucidate the existence of any functional zonations^28^. Since this work examined afferent PVS in mice, perivenous spaces should be studied and comparative anatomy of PVS should be investigated in human brains that are comprised of larger vessels. Partial anatomic separation of EPS and PAS may indicate distinct but related functions and as this study focused on PAS, the physiological changes involving EPS should be further analyzed^29–31^, since both EPS and PAS functions may likely be pertinent to brain maintenance^32,33^. Finally, additional meningovascular relationships and hydraulic resistance in heterogeneous EPS/PAS types should be investigated in future studies^34^, as they may offer knowledge regarding feasibility and efficacy of future diagnostics and therapeutics, including novel intrathecal drug delivery approaches in humans^35^.

## Methods

### Animals and tissue preparation

Experiments were performed according to NIH guidelines and protocols were approved by the University of Rochester Committee on Animal Resource (UCAR) (Protocol 2011–023). Mice were housed at ambient temperature of 70–74°F and at humidity of 30–70%. For ex vivo histologic investigation of PVS anatomy, 31 adult mice (2-month-old, both sexes; C57Bl/6J, Charles River) were anesthetized and transcardially perfused with 4% paraformaldehyde (PFA, Sigma-Aldrich). Extracted brains were carefully handled and immersion fixed in PFA for 24 h at 4 °C. For series 1 (*n* = 6), brains were postfixed in sucrose gradient (up to 30%), embedded in OCT and cryosectioned at 6 μm thickness in coronal plane. For series 2 (*n* = 6), brains were postfixed in sucrose gradient (up to 30%), embedded in OCT and cryosectioned at 6 μm thickness along oblique (left hemisphere) or axial planes (right hemisphere). For series 3 (*n* = 6), brains were blocked in the coronal plane then dehydrated, embedded in paraffin wax and serially sectioned at 6 μm thickness. For series 4 (*n* = 3), 100-μm thick coronal brain sections were generated using a Leica VT 1200S vibratome (Leica Biosystems, IL). For series 5 (*n* = 1), one mouse was perfused with Alexa Fluor 647-conjugated wheat germ agglutinin lectin (15 μg/ml in ice-cold PBS; Invitrogen) prior to sacrifice, and the extracted brain was sectioned at 100 μm in the coronal plane using a Leica VT 1200S vibratome (Leica Biosystems, IL). For series 1–5, alternate brain sections were sequentially labeled for immunohistochemistry. Additional 2-month-old adult animals were processed for cisternal tracer analysis (*n* = 6), tissue clearing analyses (*n* = 2), and ultrastructural PVS analysis (*n* = 1) as described below. In addition, age-related analysis was performed in 13-month-old animals (*n* = 6), as described, yielding a total of 37 animal subjects in the study.

### Immunofluorescence

Paraffin sections were deparaffinized and rinsed in ethanol and PBS whereas cryosections were rinsed in PBS to remove OCT. For thin section analyses, slides were batch treated with 5% goat serum (Sigma) and 0.2% Triton X-100 in PBS for 1 h at room temperature and were then incubated overnight at 4 °C with anti-ERTR7 and/or anti-plectin antibodies. For validation, three monoclonal rat anti-ERTR7 primary antibodies were used: MA1-40076 (ER-TR7; Invitrogen, Rockford, IL); NB100-64932 (ER-TR7; Novus Biologicals, Centennial, CO); and sc-73355 (ER-TR7; Santa Cruz Biotechnology, Inc., Dallas, TX). Likewise, three mouse or rabbit anti-plectin antibodies were used: sc-33649 (10F6; Santa Cruz Biotechnology, Inc., Dallas, TX); ab32528 (E398P; Abcam, Cambridge, MA); and PA5-79829 (polyclonal; Invitrogen, Rockford, IL). Data were depicted using NB100-64932 and sc-33649 antibodies. To determine the relationship of ERTR7 label with afferent cerebral vessels, co-labeling was performed using CY3-conjugated monoclonal mouse anti-smooth muscle actin (SMA) antibody (C6198, 1A4; Sigma, St. Louis, MO), rabbit anti-aquaporin 4 (Aqp4) antibody (AB3594-200, polyclonal; Millipore, Temecula, CA), and rabbit anti-pan-collagen antibody (PA1-85324, polyconal; Invitrogen, Rockford, IL). Following overnight primary antibody incubation, slides were rinsed in PBS and then incubated for 1 h with species-appropriate fluorescence-labeled secondary antibodies: donkey anti-rat Alexa Fluor 488 (A21208, Invitrogen, Rockford, IL), donkey anti-mouse (Alexa Fluor 555, A31570, Invitrogen, Rockford, IL; CY5, 715-175-150, Jackson ImmunoResearch Labs, West Grove, PA), and/or donkey anti-rabbit Alexa Fluor 555 (A31572, Invitrogen, Rockford, IL). For thick section analyses, sections were batch processed as above except primary and secondary antibodies were applied for 48 and 6 h, respectively, to ensure full penetration of tissues. All primary antibodies were used at 1:1000 dilution and all secondary antibodies were used at 1:500 dilution. Sections were mounted using ProLong Gold containing 4′,6-diamidino-2-phenylindole (Invitrogen, Thermo Fisher Scientific). Anti-ERTR7 antibodies were validated using sections of mouse lymph node and spleen, which confirmed cytoplasmic label in stromal regions^36^; omission of primary antibodies and rat anti-IgG2a antibody (02-9688, Invitrogen, Invitrogen, Rockford, IL) were used as negative controls, and confirmed sensitivity and reproducibility of ERTR7 label. Upon completion of immunohistochemistry analyses, select slides were uncoverslipped, re-stained with hematoxylin and eosin (H&E) and reimaged.

### Imaging and quantification

Slides were imaged using an epifluorescent research microscope (Nikon Eclipse Ni-U; Nikon Instruments Inc., Melville, NY) and/or confocal laser scanning microscope (Olympus FV3000; Olympus America, Inc.). Confocal images were rendered using Olympus cellSens software version 3. Afferent vessels were identified at cerebral cortical brain surfaces as thick-walled, SMA-positive cylindrical structures and were classified according to location (ventral, dorsal, and lateral). Images were acquired at ×200 and/or ×600 and datasets were processed and analyzed using Nikon Microscope Solutions Imaging Software (NIS-Elements AR Version 4.30.01) or ImageJ (U.S. National Institutes of Health, Bethesda, MD, USA, https://imagej.nih.gov/ij/). SMA and ERTR7-positive regions were digitally traced and circumferences (µm) and/or areas (µm^2^) enclosed by labeled regions of interest (ROI) within the SAS were determined for individual vessels, ensuring not to include vessel branch points. Differences in ERTR7 and SMA-outlined areas were recorded for each SAS vessel and were normalized as percentages of corresponding cross-sectional vessel area to calculate epipial space areas. To assess total area (µm^2^) of label, the number of ERTR7-positive pixels was determined in relation to each vessel after subtracting background fluorescence intensity associated with isotype control labels. ERTR7-positive areas were then normalized to ERTR7 cross-sectional areas and compared to the corresponding vessel caliber (i.e., areas in µm^2^) to determine normalized epipial thicknesses. Pial coverage style around vessels was confirmed by an experienced vascular neuropathologist. All SMA-positive vessels present in cross section at the SAS-brain margins of three levels of mouse brain (0.94, 1.28, and 1.62 mm posterior to bregma, from series 1) were included for analysis, yielding a total of 144 vessels. Vessels lacking any ERTR7 coverage were excluded from epipial thickness and epipial space analyses. MA1-40076 (anti-ERTR7 antibody, Invitrogen) labeled images were used for quantitation and illustration of the data. Stimulated emission depletion (STED) imaging was performed on select slides using an Abberior Instruments easy3D STED microscope and images were rendered in 3D using Oxford Instruments Imaris 3D version 9.5.

### Tracer administration and quantification

To understand patterns of CSF movement, adult mouse brains were examined following intracisternal infusions of an intermediate-molecular weight tracer (Texas Red conjugated to bovine serum albumin, 66 kDa, TxRd). Animals were sacrificed either 15 min (*n* = 3) or 30 min (*n* = 3) following tracer delivery into cisterna magna. For the procedures, anesthetized animals were fixed in a stereotaxic frame and a 30 G needle was connected to PE-10 tubing filled with artificial CSF into the cisterna magna^37^. Using this technique, 10 µl of CSF tracer was injected at a rate of 2 µl/min over 5 min with a syringe pump (Harvard Apparatus). Following sacrifice, extracted brains were immersion fixed in 4% PFA for 24 h at 4 °C, then sectioned into 100 μm coronal slices using a Leica VT 1200S vibratome (Leica Biosystems, IL). Thick sections were then sequentially processed for immunohistochemistry, as described above, using rat anti-ERTR7 (NB100-64932 ER-TR7; Novus Biologicals, Centennial CO), mouse anti-CD68 (MA5-16654, ED1, Invitrogen, Rockford, IL), and/or mouse anti-SMA, MA5-11547 [1A4 (asm-1), Invitrogen, Rockford, IL] primary antibodies followed by donkey anti-rat Alexa Fluor 488 (A31570, Invitrogen, Rockford, IL) or donkey anti-mouse CY5 (715-175-150, Jackson ImmunoResearch Labs, West Grove, PA) secondary immunofluorescent antibodies. Whole brain and montage images (500 mm^2^) at ventral, lateral and dorsal brain areas at 0.94, 1.28, and 1.62 mm posterior to bregma were acquired. After normalizing to isotype controls, tracer-positive areas were investigated at superficial brain regions. The number of ERTR7-positive pixels were determined in relation to penetrating arterioles to assess total area (µm^2^) of label within individual PAS. Areas of ERTR7-positive signal were then normalized to PAS areas to determine the percent of label within PAS. ED1 label was assessed in individual PAS using automated object count feature. Upon completion of immunofluorescent imaging experiments, mapped slides were post labeled with CY3-conjugated mouse anti-SMA antibody (C6198; Sigma, St. Louis, MO) and were reexamined to ensure that analyzed vessels excluded any venous structures.

### Mouse brain clearing, staining, and imaging

For enhanced volumetric (3D) immunofluorescent PVS imaging, two mice were processed using SHIELD protocol^38^. Following anesthetization, the animals were perfused with Alexa Fluor 647-conjugated wheat germ agglutinin lectin (15 μg/ml in ice-cold PBS; Invitrogen) and TxRd was injected into cisterna magna (0.5% in 10 µl aCSF at 2 µl/min) of one animal. Thirty minutes following introduction of fluorescent tracer(s), the animals were perfused with 4% PFA and then sacrificed. Extracted brains were cleared for several days with SmartClear II Pro (LifeCanvas Technologies), a device based on stochastic electrotransport^39^. Cleared brains were then actively immunolabeled within 24 h using SmartLabel (LifeCanvas Technologies), a device based on eFLASH technology^40^ that integrates stochastic electrotransport^39^ and SWITCH^41^. For processing, the following primary antibodies were used: anti-ERTR7, NB100-64932 (ER-TR7; Novus Biologicals, Centennial, CO) and anti-SMA, MA5-11547 [1A4 (asm-1), Invitrogen, Rockford, IL]. Species appropriate fluorescently conjugated secondary antibodies were applied in 1:2 primary:secondary molar ratios (A21208, Invitrogen, Rockford, IL; 715-175-150, Jackson ImmunoResearch, West Grove, PA). Samples were next refractive index matched through incubation in EasyIndex (LifeCanvas Technologies) and then imaged at ×15 with a SmartSPIM light sheet microscope (LifeCanvas Technologies), and images were rendered in 3D using Oxford Instruments Imaris 3D version 9.5.

### Transmission electron microscopy (TEM)

To analyze PVS ultrastructural anatomy, one mouse (*n* = 1) was processed for transmission electron microscopy (TEM). The animal was transcardially perfused with 2.0%/2.5% PFA and glutaraldehyde solution in 0.2 M sucrose with 0.1 M sodium cacodylate (pH 7.4). Following brain extraction and fixation, coronal brain sections (100 µm) were prepared and post-fixed in 1.0%/1.5% osmium tetroxide and potassium ferrocyanide. Sections were rinsed in distilled water then dehydrated in graded alcohol and transitioned into propylene oxide/resin (1:1) followed by 100% resin. Subsequently, the sections were placed between coated glass slides (SigmaCote, Sigma/Aldrich, St. Louis, MI) for 24 h at 60 °C to allow for polymerization. Arteries, arterioles and superficial cerebral cortical tissues were isolated from embedded samples and mounted onto blank epoxy blocks. Samples were sectioned at 1 μm thickness using an ultramicrotome, mounted on glass slides and stained with 1% toluidine blue. Following verification of vessel type by light microscopy, 70 nm ultrathin sections were prepared, loaded into formvar/carbon coated nickel slot grids and imaged using a Hitachi 7650 transmission electron microscope and Gatan 11 megapixel Erlangshen digital camera for digital capture using Digitalmicrograph software.

### Immuno-electron microscopy (immuno-EM)

To supplement TEM and to localize the ERTR7 label, immunoelectron microscopy (Immuno-EM) labeling was performed on two additional mouse brain specimens (*n* = 2) using a pre-embedding technique performed on coronal 100 µm-thick brain sections collected from series 4. Samples were permeabilized in 0.1% Triton X-100 with 5% goat serum (Sigma) for 2 h and then incubated for two days at 4 °C with a monoclonal rat ERTR7 antibody (NB100-64932, Novus Biologicals, Centennial CO, 1:1200). Following PBS rinse, samples were incubated overnight at 4 °C in preadsorbed biotin goat anti-rat secondary antibody (Ab7096, Abcam, Cambridge, UK, 1:200). Then they were rinsed and incubated in the dark in ExtrAvidin Peroxidase (2886, Sigma, 1:100) for 90 min, rinsed again in PBS and TRIS buffer and pre-soaked in 0.6% diaminobenzidine (DAB)/TRIS (25 min) and treated with DAB/hydrogen peroxide (0.03%) solution (8 min). Following additional rinses in Tris and 0.1 M sodium cacodylate buffer, sections were fixed overnight at 4 °C in 2.5% glutaraldehyde in 0.1 M sodium cacodylate buffer. After further rinsing in 0.1 M sodium cacodylate buffer and double distilled water, the sections were incubated at 60 °C in a 0.2% silver nitrate solution (10 min) to intensify DAB label. They were then rinsed in ddH_2_O followed by 0.05% gold chloride, ddH_2_O, aqueous 2.5% sodium thiosulfate, and ddH_2_O before being post-fixed in 1.0% osmium tetroxide in 0.1 M sodium cacodylate buffer (30 min). After final rinse in buffer and then ddH_2_O, they were processed as described above for TEM imaging.

### Aged mice, APP/PS1 mice, and methoxy-X04 analysis

To investigate PVS changes associated with aging and cerebral amyloid angiopathy (CAA), double transgenic mice expressing a chimeric mouse/human amyloid precursor protein (Mo/HuAPP695swe), and a mutant human presenilin 1 (PS1-dE9) both directed to CNS neurons were also investigated along with wildtype littermate controls. The APPswe/PS1dE9 (APP/PS1) mice and wildtype controls, 13-month-old, regarded as middle-aged and referred to here as “old” (*n* = 3 per group; male and female) were purchased from Jackson Laboratory (Bar Harbor, ME, USA)^5^. The animals were anesthetized with a mixture of ketamine (100 mg/kg, IP) and xylazine (10 mg/kg, IP). For visualization of plaques and cerebrovascular amyloid, methoxy-X04 (MeX04, Tocris Bioscience, Bristol, UK) dissolved in DMSO (10%), propylene glycol (45%), and PBS (45%) was administered (IP; 10 mg/kg). Twenty-four hours after MeX04 administration, animals were perfused with Alexa Fluor 647–conjugated wheat germ agglutinin lectin (15 μg/ml in ice-cold PBS; Invitrogen) and TxRd was injected intracisternally (0.5% in 10 µl aCSF, injected at 2 µl/min). Thirty minutes following introduction of fluorescent tracers, the animals were perfused with 4% PFA and then sacrificed.

Following brain extractions, 100 µm-thick coronal brain sections were generated via vibratome sectioning. Thick sections were batch processed by blocking with 5% goat serum (Sigma) and 0.2% Triton X-100 in PBS for 2 h at room temperature, incubating for 48 h at 4 °C with anti-ERTR7 (NB100-64932, Novus Biologicals, Centennial, CO) and/or mouse anti-ED1 (anti-CD68, MA5-16654, Invitrogen, Rockford, IL) primary antibodies, then rinsed in PBS and incubated for 6 hr with species-appropriate fluorescence-labeled secondary antibodies (donkey anti-rat Alexa Fluor 488; Invitrogen/Molecular Probes, or donkey anti-mouse Texas Red; Invitrogen/Molecular Probes). Images were acquired at ×200 and/or ×600. ROI of uniform dimensions, incorporating sites of arteriolar penetration were acquired for individual vessels in ventral, lateral, and dorsal brain regions. To assess total area (µm^2^) of label, the number of ERTR7-positive pixels was determined in relation to each vessel after subtracting background fluorescence intensity associated with isotype control labels and pial coverage styles around vessels were confirmed by an experienced vascular neuropathologist. For acquisition, slides were imaged using an epifluorescent research microscope (Nikon Eclipse Ni-U; Nikon Instruments Inc., Melville, NY) and/or confocal laser scanning microscope (Olympus FV3000; Olympus America, Inc.). Upon completion of immunofluorescence imaging and analysis, mapped slides were post labeled with CY3-conjugated mouse anti-SMA antibody (C6198; Sigma, St. Louis, MO) and were reexamined to ensure that analyzed vessels excluded venous structures. All vessels with thick SMA-positive layers present in cross section at the SAS-brain margins of three levels of mouse brain (0.94, 1.28, and 1.62 mm posterior to bregma, from series 1) were included for analysis. Stimulated emission depletion (STED) imaging was performed on select slides using an Abberior Instruments easy3D STED microscope and images were rendered in 3D using Oxford Instruments Imaris 3D version 9.5.

### Cranial window surgery and in vivo two-photon laser scanning microscopy

To investigate the physiology in live mice, in vivo imaging was performed in 8–12-week-old male mice C57BL/6 mice (Charles River Laboratories) (*n* = 8). Animals were anesthetized with a combination of ketamine (100 mg/kg) and xylazine (10 mg/kg) administered intraperitoneally. Body temperature was kept at 37 °C with a temperature-controlled warming pad. Depth of anesthesia was determined by the pedal reflex test. Once reflexes had ceased, anesthetized mice were placed in a stereotaxic frame and head-fixed for the surgical procedure. A craniotomy (~4 mm in diameter) was then performed over the right MCA vascular territory. The dura mater was removed, and the craniotomy was filled with agarose (1% at 37 °C), covered with a glass coverslip, and sealed with dental acrylic. A 30GA needle was inserted into the cisterna magna for intracisternal tracer injections^37^. The dura mater of mice was exposed after blunt dissection of the neck muscles so that a cannula could be implanted into the CM, which is continuous with the subarachnoid space. To visualize tracer movement from the subarachnoid space of the cisterna magna into the periarterial spaces, Texas Red-dextran 70 (MW 70 kDa; 0.5% in aCSF, Invitrogen) was injected intracisternally at a rate of 2 µl/min for 5 min immediately before imaging.

A Chameleon Ultra II laser (Coherent) attached to a resonant scanner Bergamo scope (Thorlabs) was used for in vivo two-photon imaging. A ×20 (1.0 NA, Olympus) water immersion lens was used to acquired time-lapse Z-stacks from the surface of the brain to a depth of 100–150 µm. An excitation wavelength of 890 nm was used to produce second harmonic generation (SHG) and excite a Texas Red labeled dextran. Two color channels were collected simultaneously (447 nm blue and 607 nm red in GaAsP detectors). The 447-nm channel captured SHG from collagen, while the 607-nm channel captured the intracisternal Texas Red-dextran. The images obtained were 16-bit with spatial dimension of 512 by 512 pixels.

For each animal, four to seven penetrating arterioles were distinguished on the basis of morphology: surface arteries passing superficially to surface veins and exhibiting less branching at superficial cortical depths. For evaluation of the PAS type, the SHG from collagen was first imaged by averaging 10 frames per *Z*-plane to a final frame rate of 1.5 Hz. For tracer movement along the PAS, the penetrating arterioles were repeatedly scanned from the surface to 100–150 µm below the surface with 1 µm z-steps at ~10 min intervals for the duration of the experiment (~60 min).

Image analysis was conducted using ImageJ software (NIH), Matlab (MathWorks), and IMARIS (v. 9.7, Bitplane, Concord, MA, USA). Image registration via rigid translation was performed on each image in the time series. The average channel pixel intensity due to noise within the PAS prior to the intracisternal injection was subtracted from each subsequent time point post-injection per mouse. To determine the presence of CSF tracer at the imaging planes, one brightness threshold was chosen per animal such that the pixel intensity was at least 10% of the maximum intensity captured in the imaging. The maximum depth of CSF tracer appearing and the time between each image in the time series were considered to calculate the rate at which PAS fills with dye postinjection.

### Statistical analysis

Statistical analyses were performed using Prism 8 (GraphPad Software, Inc., La Jolla, CA). The statistical test used in each analysis is stated in the figure legend. Statistical tests were selected after evaluating the distribution of each dataset and assessing normality using a Smirnov–Kolmogorov tests. All *P* values were 2-tailed using a level of 0.05 as the criterion for statistical significance.

### Statistics and reproducibility

Where possible, exact *p* values are provided. The statistical software used for this study (Prism 8; GraphPad Software, Inc., La Jolla, CA) is unable to provide exact values for *P* < 0.0001. Immuno-EM images are representative of six brain sections from two mice. For all other images, data are representative of at least three sections from different anatomical regions, and from at least 3 mice per group.

### Reporting summary

Further information on research design is available in the Nature Research Reporting Summary linked to this article.

## Supplementary information

Supplementary Figures

Reporting Summary

Description of Additional Supplementary Files

Supplemental Movie 1

Supplemental Movie 2

Supplemental Movie 3

Supplemental Movie 4

Supplemental Movie 5

Supplemental Movie 6

Supplemental Movie 7

Supplemental Movie 8

Supplemental Movie 9

Supplemental Movie 10

Supplemental Movie 11

Supplemental Movie 12

Supplemental Movie 13

## Data Availability

The data supporting the findings from this study are available within the manuscript and its supplementary information. Source data are provided with this paper.

## Source data

Source data
