# Supplementary material for: Periarteriolar spaces modulate cerebrospinal fluid transport into brain and demonstrate altered morphology in aging and Alzheimer’s disease
Source: Nat Commun. 2022 Jul 6;13:3897. doi: 10.1038/s41467-022-31257-9 (PMC9259669; doi:10.1038/s41467-022-31257-9)
Supplement: Supplementary file 1 — Supplementary Figures [file 41467_2022_31257_MOESM1_ESM.pdf]

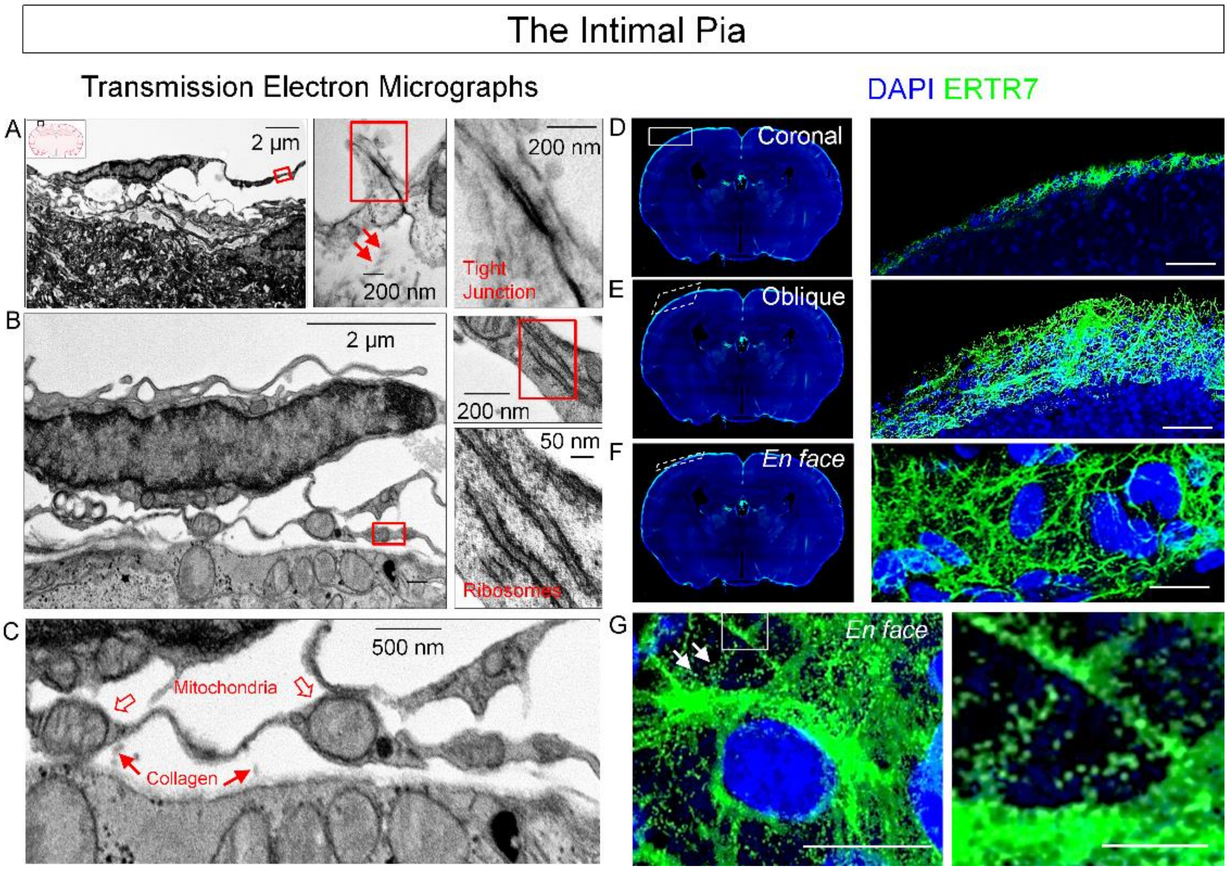

Supplementary Figure 1. Intimal pial cells form an interconnected, reticulated lace-like sheath along the surfaces of cortical mouse brain. Ultrastructural images of intimal pial cells demonstrate their morphology, with elongated processes emanating from thin perinuclear cytoplasmic rims (A,B). Higher power images of boxed areas in (A) demonstrate an intercellular tight junction between adjacent pial cells. Higher power images of boxed areas in (B) demonstrate inter-mitochondrial ribosomes, intermediate filaments and rough endoplasmic reticulum. An enlargement of B is shown in (C) and depicts cell processes with irregular cytoplasmic accumulations and mitochondria that produce a beaded appearance. Collagen fibrils are seen next to pial cells, but basal laminae are not observed (C). ERTR7 immunohistochemistry further delineates the reticulated cytology of intimal pial cells at the brain surface, as shown on images of coronal (D), oblique (E), and axial *en face* (F) superficial mouse brain sections. A cropped image from F is shown in (G) and depicts an isolated pial cell with innumerable cytoplasmic processes (white arrows) and beaded appearance (G, inset). (D-G) Green/FITC, ERTR7; blue, DAPI; scale bars = (D,E) 100 µm; (F,G) 10 µm; (G, right/inset) 1 µm. Epifluorescent images: D-F left-hand side. Confocal images: D-F right-hand side and G. TEM data are representative of 92 images from 3 young mice, encompassing ventral, lateral, and dorsal brain regions.

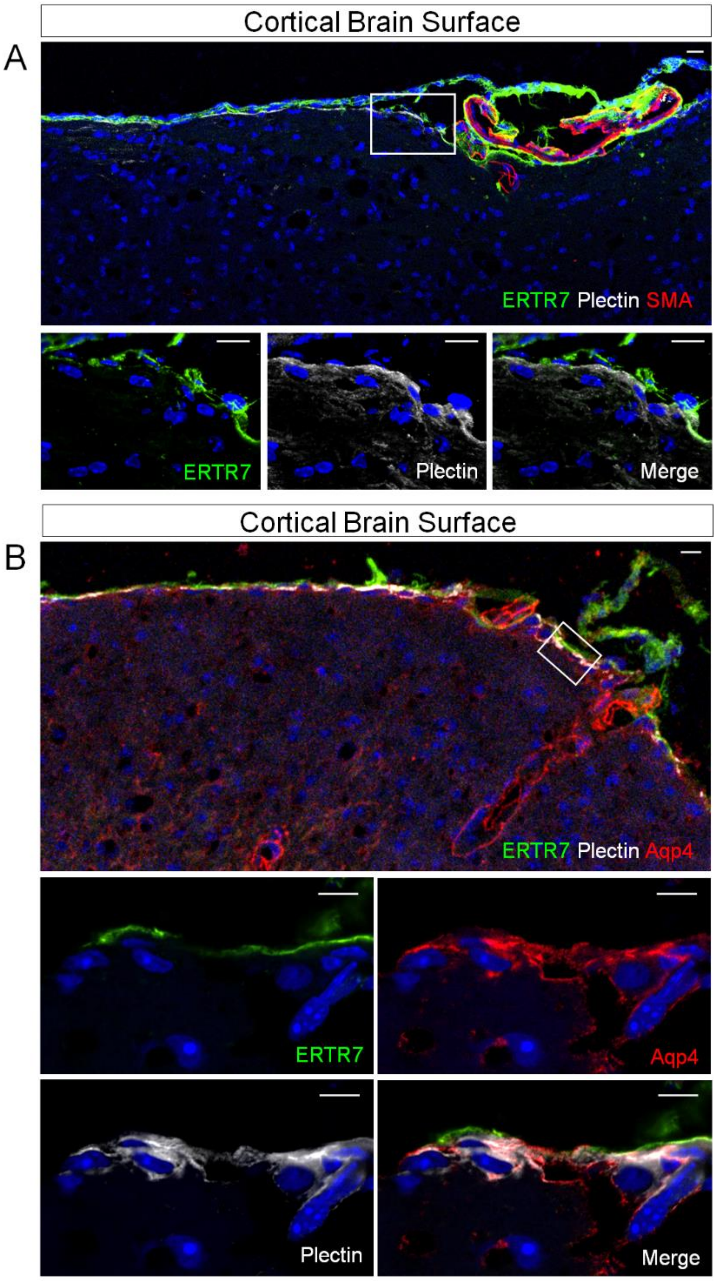

Supplementary Figure 2. Plectin co-labels astrocytes (i.e., glia limitans) at superficial mouse brain regions. Coronal section of superficial dorsal mouse brain co-labeled with ERTR7 and plectin demonstrate labeling of distinct cell populations (A). Images of brain sections triple labeled with aquaporin 4 suggest that the plectin-positive cells are astrocytes of the glia limitans (B). Red/CY3, SMA or aquaporin 4; green/FITC, ERTR7; white/CY5, plectin; blue, DAPI. Scale bars = (A and B, upper panels) 10 μm; (A and B, lower panels) 5 μm. Images are depicted using sc-33649 mouse anti-plectin antibody (Santa Cruz Biotechnology, Inc.). Data are representative of 154 sections from 3 young mice, encompassing ventral, lateral, and dorsal brain regions.

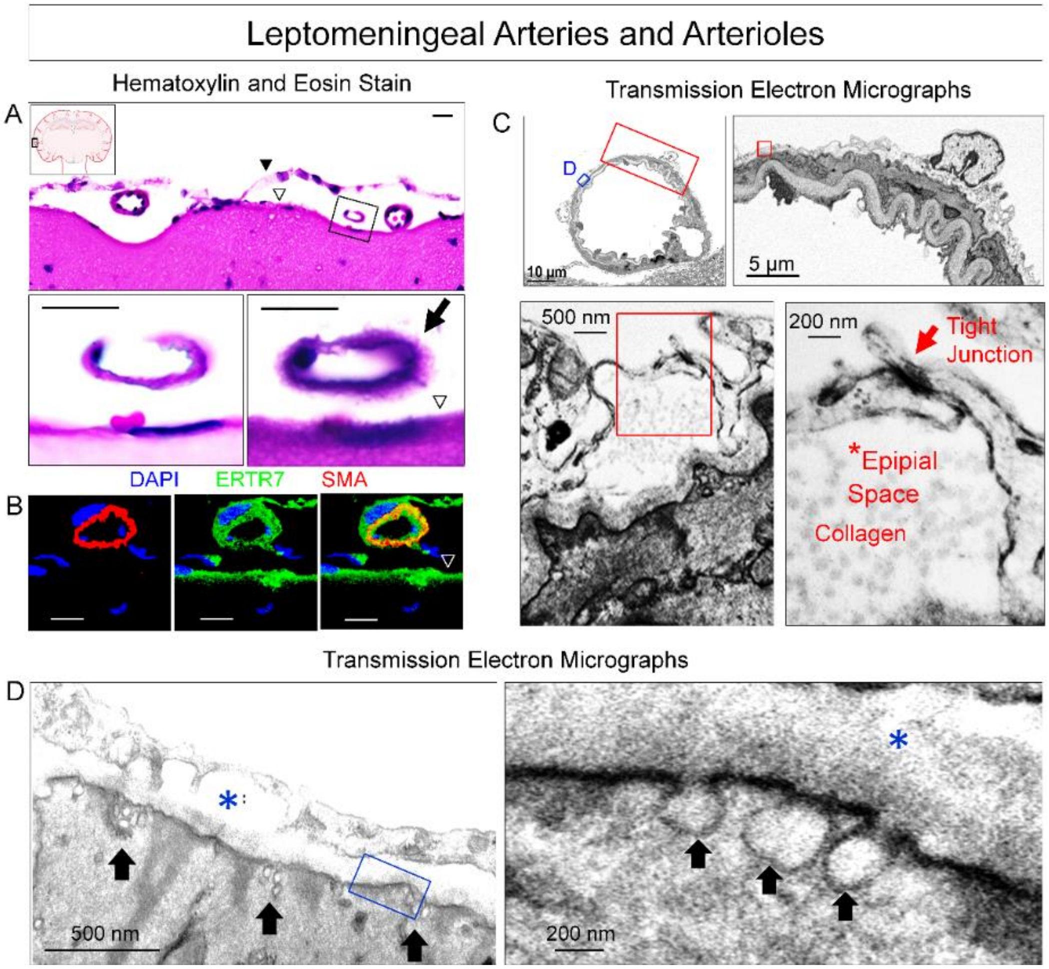

Supplementary Figure 3. The epipia attenuates and forms a reticulated, lace-like sheath along small cerebral arteries and arterioles. Routine coronal section of lateral mouse brain demonstrates small caliber leptomeningeal arteries (A). Enlargement of the boxed vessel, shown in different focal planes (A, lower panel), highlights the irregular shaggy adventitial border constituting the reticulated epipia (black arrow). At this level, the ERTR7-positive epipial layer forms a thin, lacy network that variably infiltrates the tunica media (B). The arachnoid and intimal pia are marked by solid and/or open arrowheads, respectively (A,B). A micrograph of a small artery is shown (C, upper left image). As depicted in higher power micrographs of red boxed areas (C), tight junctions adjoin the processes of neighboring epipial cells (red arrow). Higher magnification of the blue boxed area in C is shown (D) along with enlargement of a cropped region. Notice that pinocytotic vesicles (D, black arrows) are present on the abluminal aspect of vascular smooth muscle cell surfaces that border the epipial space. Loosely packed collagen fibrils are seen within the epipial space (asterisks) without distinct basal laminae. (A) H&E; (B) Red/CY3, SMA; green/FITC, ERTR7; blue, DAPI; scale bars = (A-C) 10 μm (C,D) transmission electron micrographs with scale bars as indicated. TEM data are representative of 50 vessels from 3 young mice.

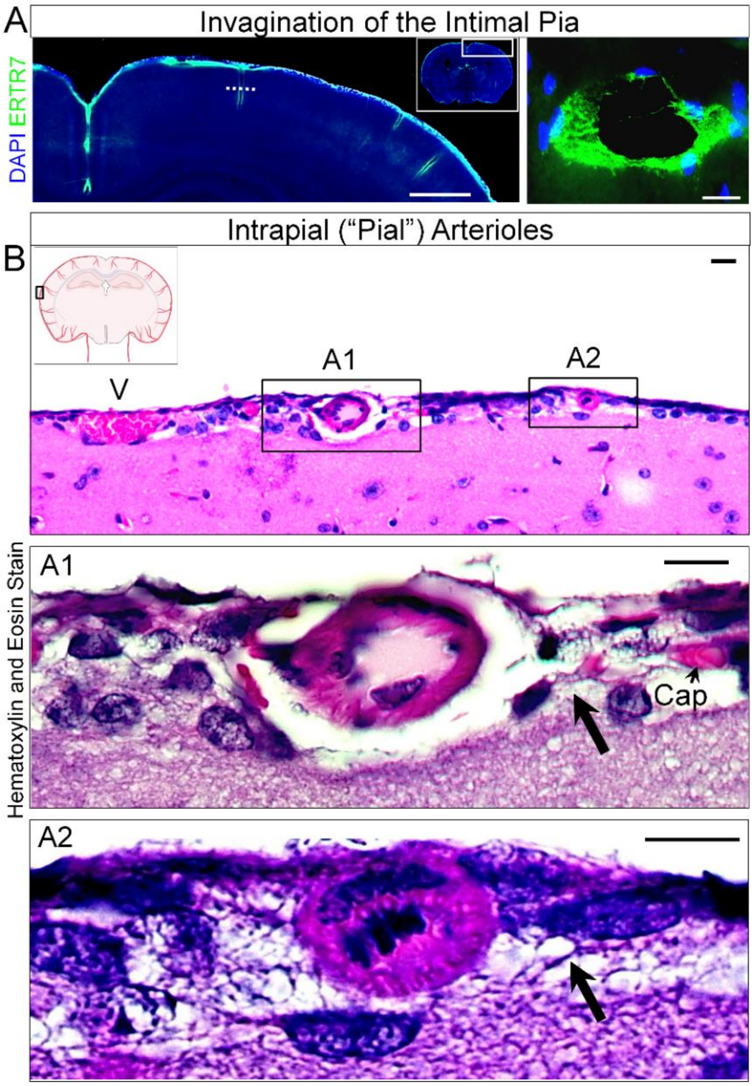

Supplementary Figure 4. The epipia and intimal pia intermingle at the brain surface, creating a space or a variably dense, net-like funnel around penetrating arterioles. As shown on coronal section (A) and axial section (B), which represents the area by dotted line in A, the intimal pia invaginates around penetrating arterioles within cortical brain. Routine section from lateral brain region illustrates intrapial arterioles (i.e., A1 and A2). Enlargement of the boxed areas highlight loosely packed pial cell processes (arrows) that form perforated funnels that are patent or partially covered at sites of cerebral penetration. Depictions represent appearances of type B and type C periarteriolar vessels, as defined in Figure 3. (A) Red/CY3, SMA; green/FITC, ERTR7; blue, DAPI; (B) H&E; scale bars = (A, left) 500 µm; (A, right and B) 10 µm; Abbreviations: Cap, intrapial capillary; V, intrapial venule. Pial anatomy depicted is representative of approximately 1000 vessels from 18 young mice.

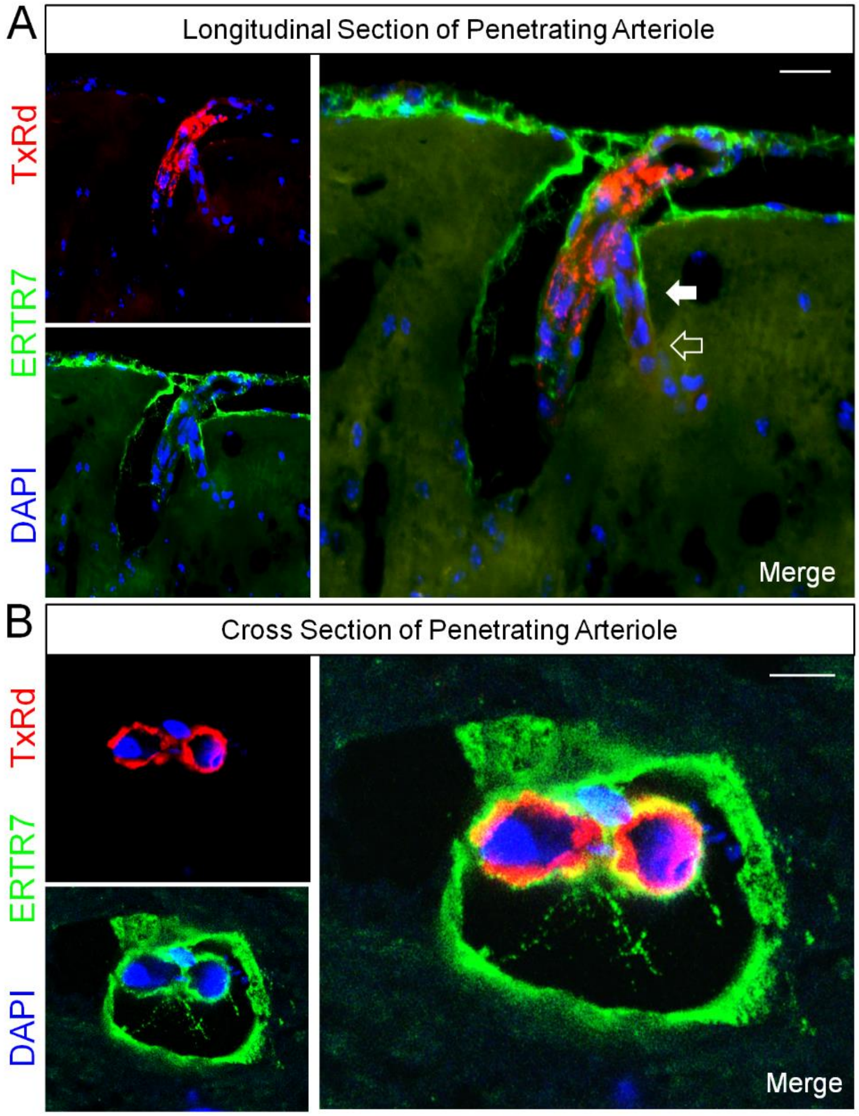

Supplementary Figure 5. Penetrating arterioles are often eccentrically positioned within PAS due to irregularity of secondary arteriolar branches. Coronal superficial dorsal brain section demonstrates a penetrating arteriole tethered to one side of a PAS, due to emergence and penetration of a secondary arteriolar branch (solid arrow) (A). Note the beaded, ERTR7-positive pial cell elements within the PAS. Loss of ERTR7 ensheathment on the secondary arteriole branch is appreciated (open arrow) shortly after its brain penetration (solid arrow), wherein the vessel transitions into a capillary structure. Cross section of a separate vessel, similar to the level of the solid arrow in A is shown in (B), and further illustrates anatomic relationships at the site of arteriolar penetration. Depictions represent a Type C PAS, as defined in Figure 3. (A,B) Red/CY3, SMA; green/FITC, ERTR7; blue, DAPI. scale bars = (A,B) 10 μm. Pial anatomy depicted is representative of approximately 1000 vessels from 18 young mice.

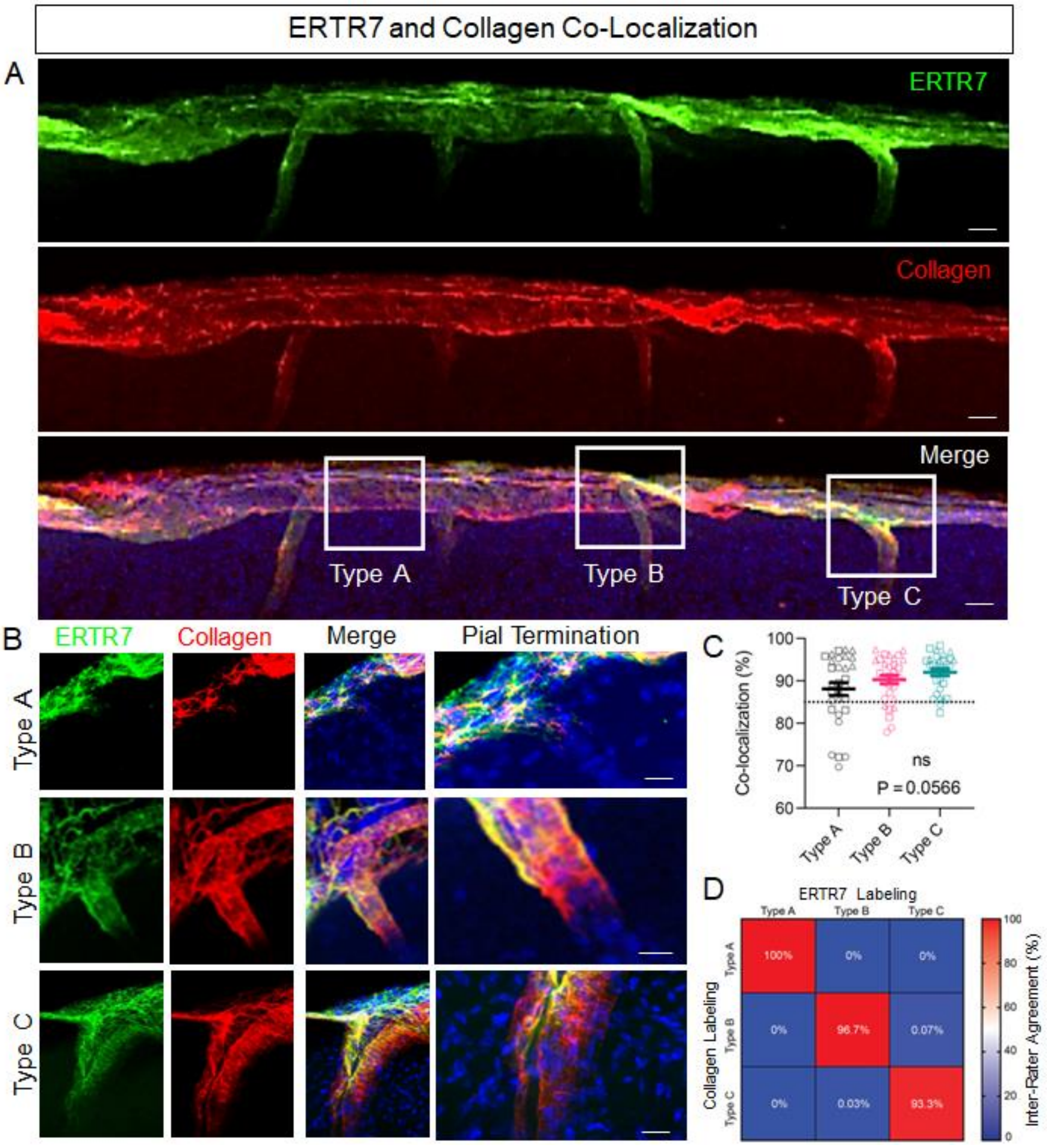

Supplementary Figure 6. ERTR7 and collagen co-localize within pia mater of mouse brain. Coronal section of dorsal mouse brain demonstrates ERTR7 and collagen co-localization at the superficial cerebral cortical brain region and around several penetrating arterioles including type A, type B, and type C vessels (A). Cropped images of type A, type B, and type C arterioles demonstrate clear delineation of the vessel types (B). The percent of co-localized pixels of both labels is shown in type A, B, and C vessels (C). One way ANOVA, P 0.0566, ns: not significant. (D) Intra-rater agreement of PAS typing using ERTR7 and collagen labels. Overall concordance was 97% for all three PAS types, with only 3 out of 90 vessels differing between labels (0.03%). (A,B) Red/CY3, collagen; green/FITC, ERTR7; blue, DAPI. n=90 vessels (30 PAS per type) obtained from 8 animals; scale bars = (A) 30 μm, B) 10 μm. Pial anatomy depicted is representative of approximately 1000 vessels from 18 young mice.

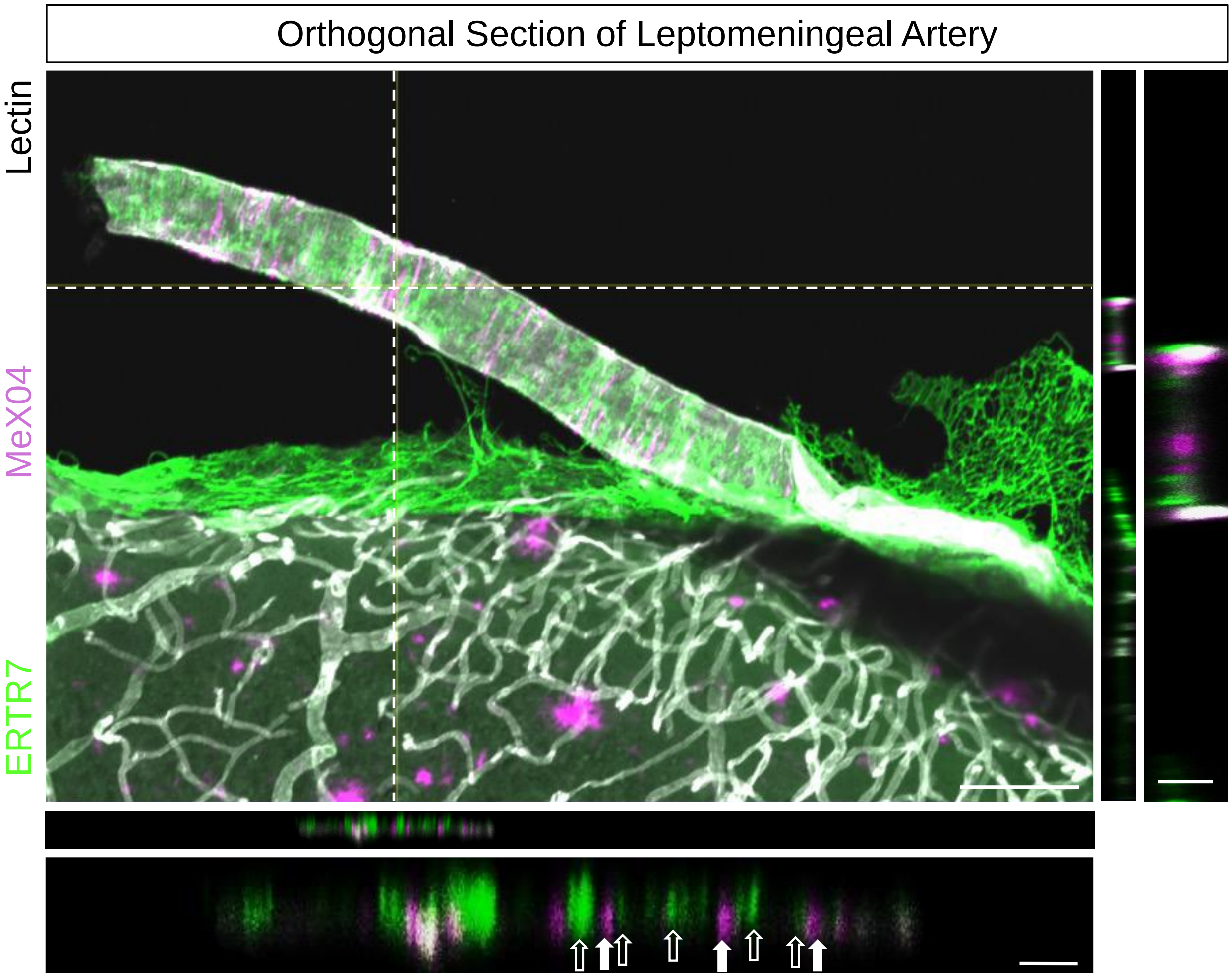

Supplementary Figure 7. Leptomeningeal vessel from an old APP/PS1 mouse brain shown with MeX04 label. Epipial and intimal pial linings are depicted around a leptomeningeal artery present at the cerebral cortical brain surface of an old APP/PS1 mouse brain. Note in orthogonal images, present with enlargements at bottom and right-hand side, that MeX04 label (closed arrows) appear within the epipial space and is enmeshed within the ERTR7-positive cell processes (open arrows). Green/FITC, ERTR7; magenta, MeX04; white, lectin. Scale bars = (A) 100 μm in main image; (B) 20 μm in orthogonal insets. MeX04 deposition pattern depicted is representative of 76 vessels viewed in orthogonal plane, from 3 old mice.

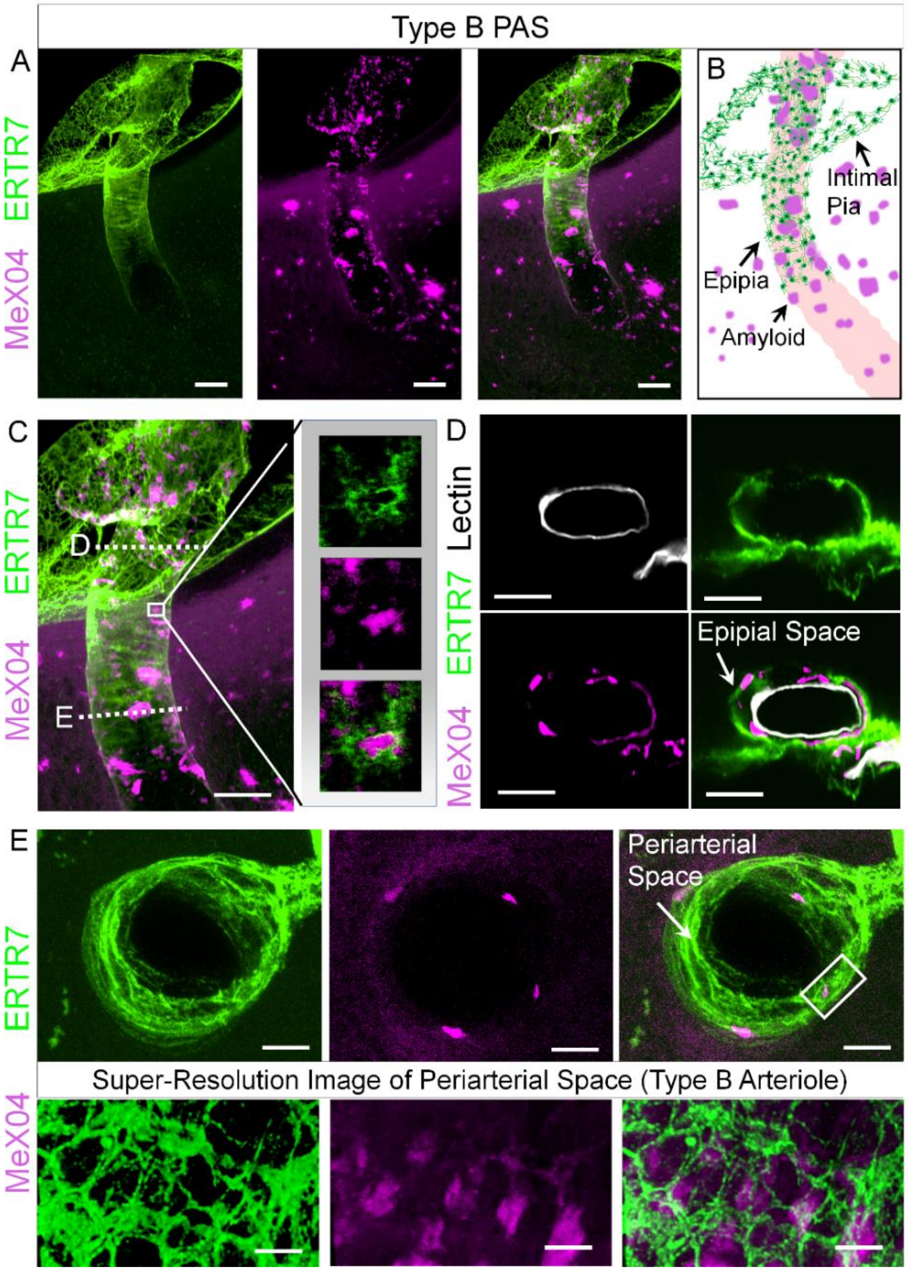

Supplementary Figure 8. Type B PAS from an old APP/PS1 mouse brain shown with MeX04 label. A periarteriolar space with merged dual pial membranes (i.e., intimal pia and epipia) is shown from cortical region of APP/PS1 mouse brain (A). Pial structures and relationships to amyloid-β deposits are depicted in the schematic (B). Cropped image (C) demonstrates amyloid-β embedment within the pial cells, as shown to advantage in inset on right-hand side. Cross sections of a leptomeningeal arteriole (D) and a penetrating arteriole, which represent areas by dotted lines in (C) are shown and depict amyloid-β within the pial layers as well as within the epipial and periarteriolar spaces. Super-resolution image of the pial network within a periarteriolar space is shown at higher magnification in the inset (E, lower panel). Green/FITC, ERTR7; violet/CY5, MeX04. Scale bars = (A,C-E) 10 μm; (E, inset) 1 μm. MeX04 deposition pattern depicted is representative of 50 Type B vessels from 3 old mice.

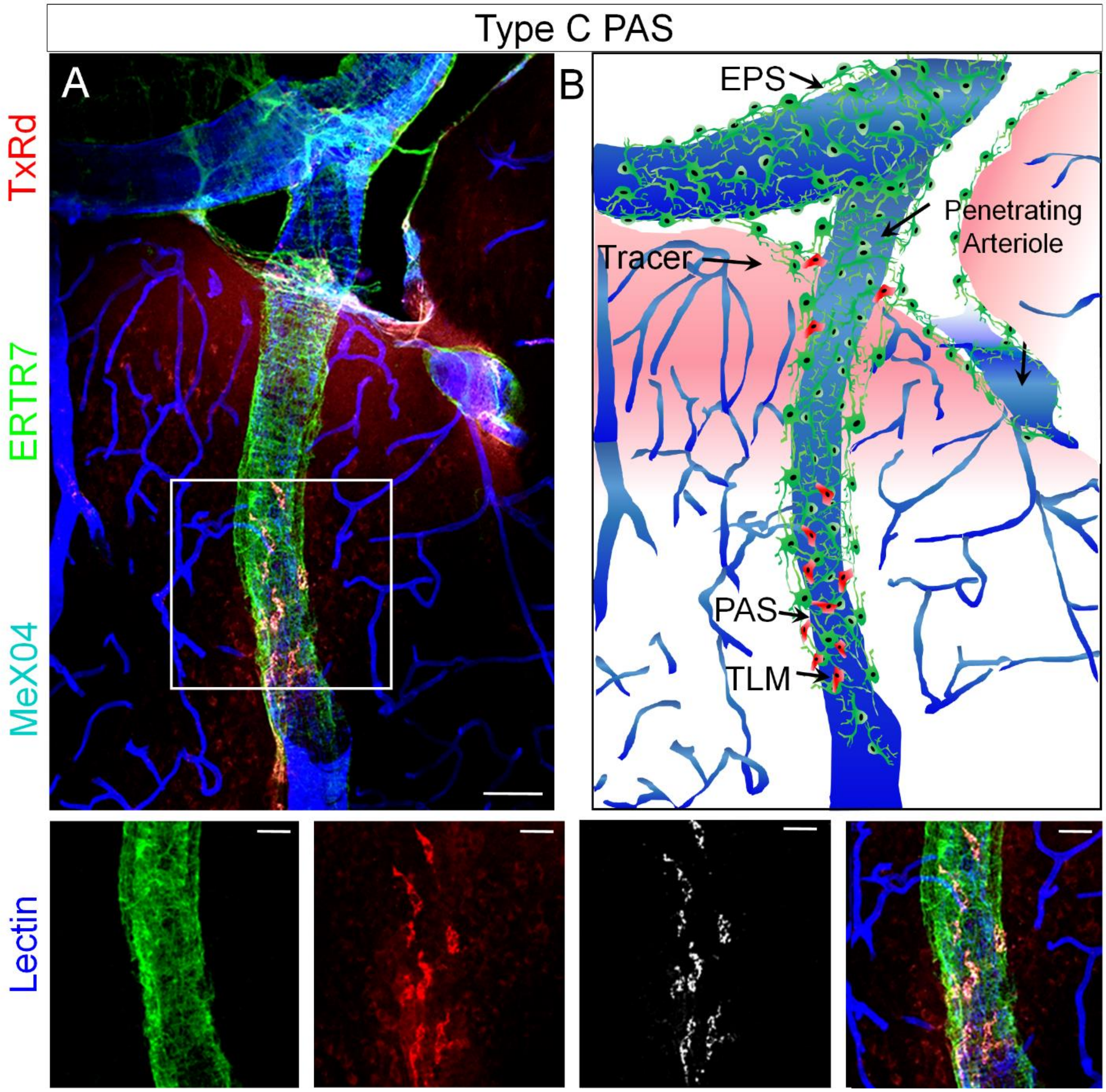

Supplementary Figure 9. Type C PAS from a young wildtype mouse brain shown with tracer and ED1 macrophage label. A periarteriolar space with unmerged dual pial membranes (i.e., intimal pia and epipia) is shown from cortical region of healthy young mouse brain 30 minutes following TxRd infusion into cisterna magna (A). The pial structure and relationships to accumulated tracer and macrophagic cells are depicted in the schematic (B). Macrphages at the brain surface are rare, however cropped image (C) demonstrates several macrophages deep within the periarteriolar space (A, lower panel). Blue, Lectin; green/FITC, ERTR7; red/Texas Red, TxRd; white/CY3, ED1. Scale bars = (A, upper panel) 50 μm; (A, lower panel) 5 μm. Type C macrophage pattern depicted is representative of 100 vessels from 9 mice.

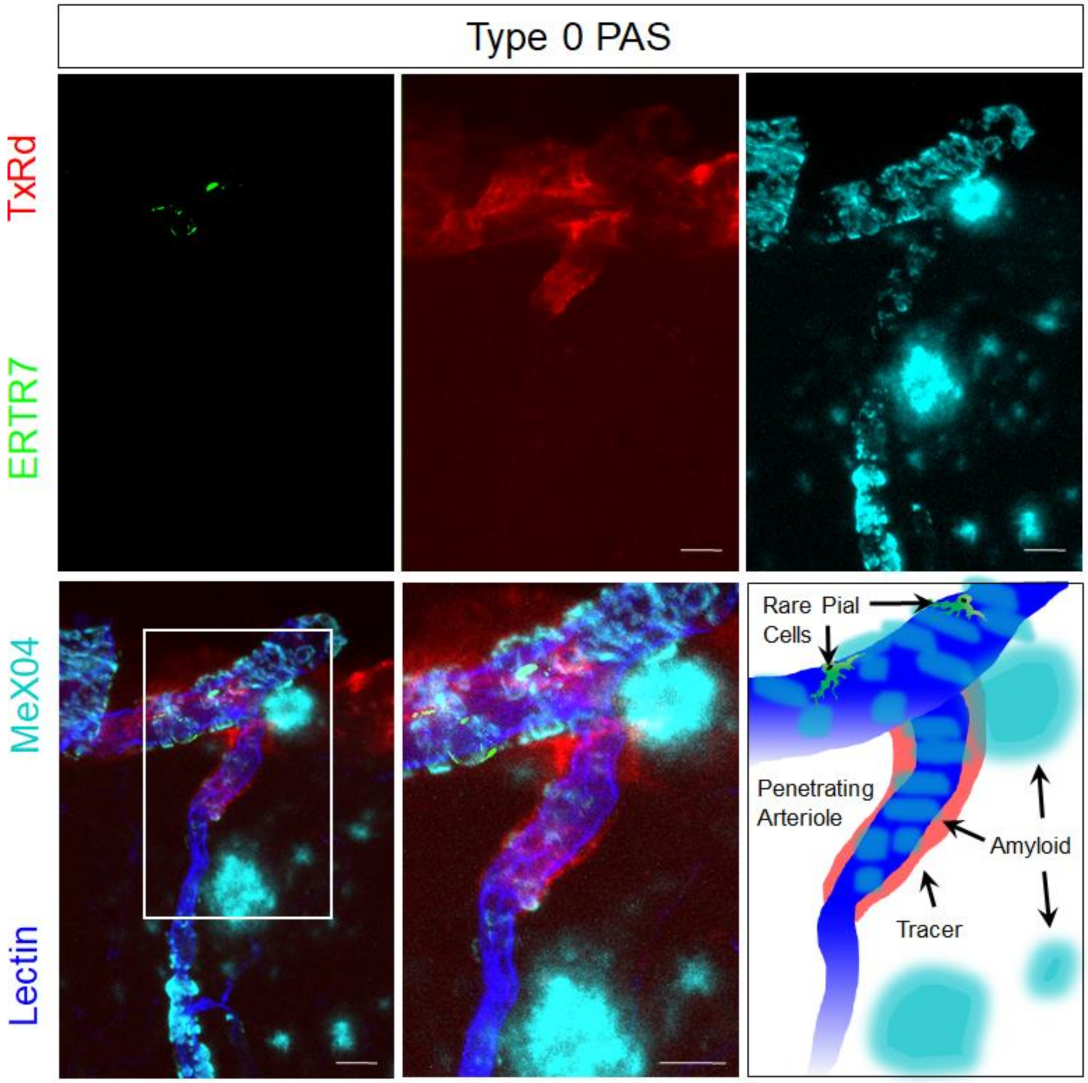

Supplementary Figure 10. Type 0 PAS from an old APP/PS1 mouse brain shown with tracer and MeX04 label. A disordered periarteriolar space with absence of pial layers is shown from cortical region of APP/PS1 mouse brain. An enlargement of the cropped area is shown in the lower panel with schematic depiction on right hand side. Note amyloid-β deposits adherent to the arteriolar walls and present within the periarteriolar space. Cyan/CY5, MeX04; Blue, lectin; Green/FITC, ERTR7; Red/Texas Red, TxRd. Scale bars = (A) 30 μm; (C) 10 μm. MeX04 deposition pattern depicted is representative of 97 Type 0 vessels from 6 old mice.

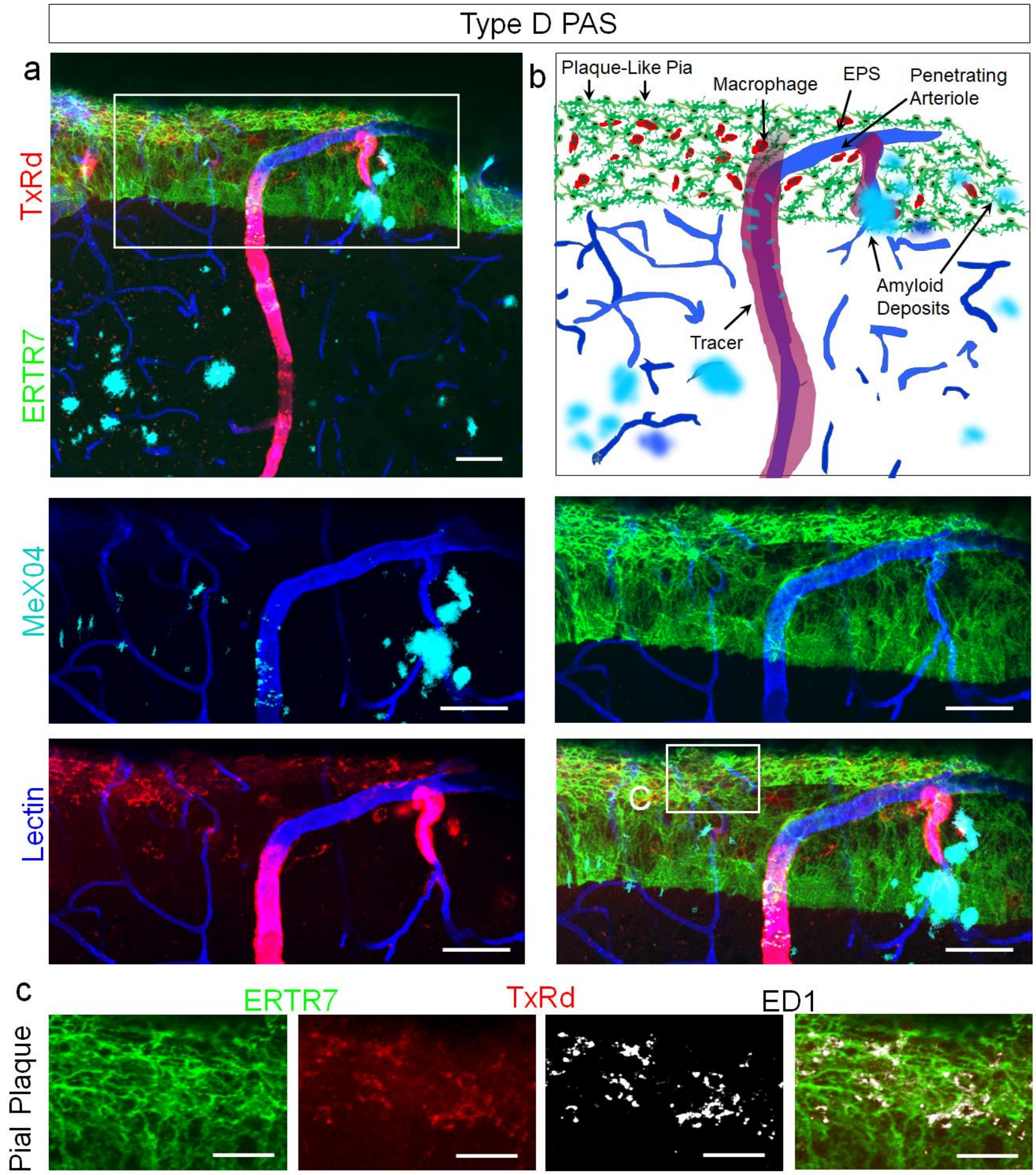

Supplementary Figure 11. Type D PAS from an old APP/PS1 mouse brain shown with tracer, MeX04 and ED1 macrophage labels. A periarteriolar space with superficial plaque-like pia is shown from cortical region of APP/PS1 mouse brain (A). Note that dual pial membranes are not identified. The pial structures and relationships to macrophages and amyloid deposits are depicted in the schematic (B). Cropped image (A, lower panel) demonstrates macrophages and amyloid-β embedment within the superficial pial plaque. Superficial (surface) macrophages are frequent and shown to advantage in higher-power inset in (C). However, note that periarteriolar macrophages are rare. Cyan/CY5, MeX04; Blue, lectin; Green/FITC, ERTR7; Red/Texas Red, TxRd; White/CY3, ED1. Scale bars = (A) 30 µm; (C) 10 µm. MeX04 deposition pattern and macrophage pattern depicted are representative of 101 Type D vessels from 6 old mice.

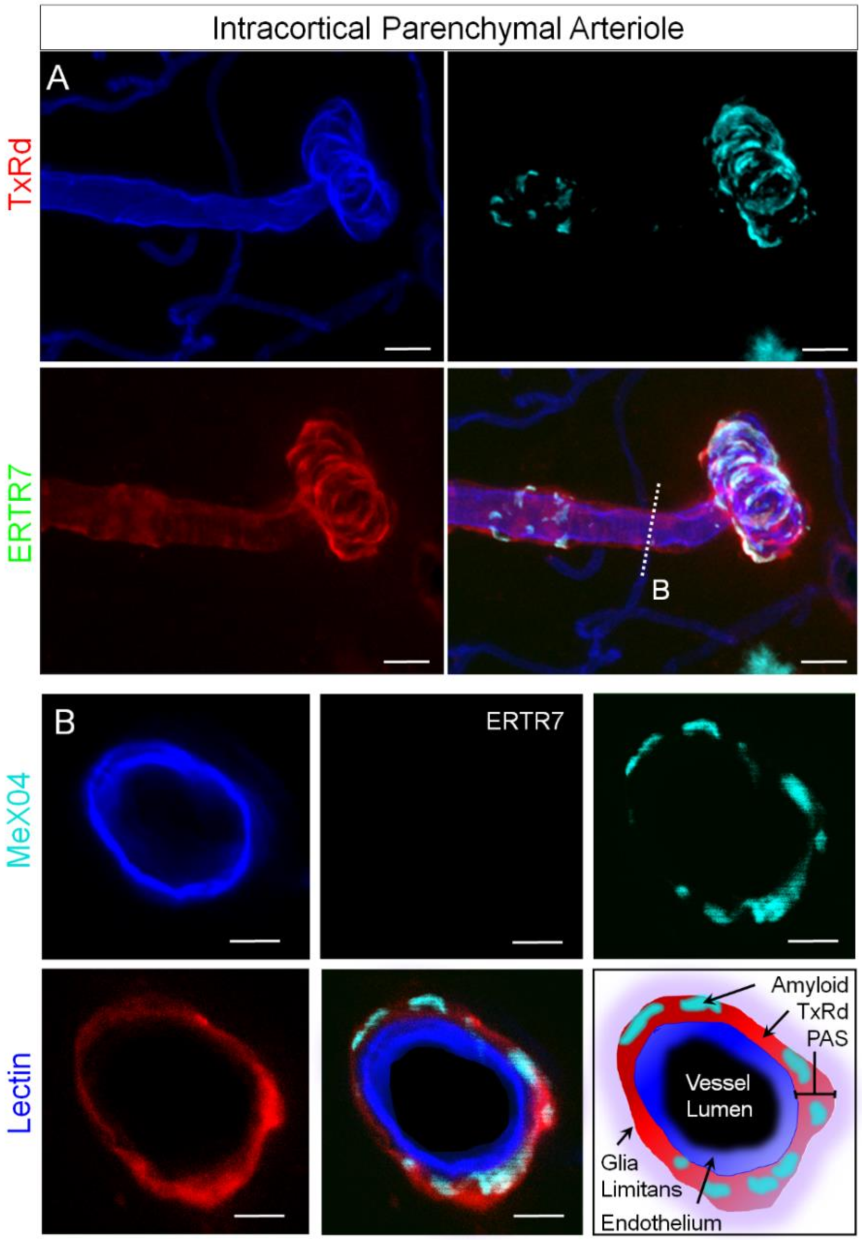

Supplementary Figure 12. Intracortical PAS from an old APP/PS1 mouse brain shown with tracer and MeX04 label. A perivascular space within deep cerebral cortex of old APP/PS1 mouse brain is shown 30 minutes following TxRd infusion into cisterna magna (A). Note that tracer conforms to amyloid in the PAS and may take on “spiral” appearance along coiled portions of the arteriole. Cross sectional image (B) of an intraparenchymal arteriole from area similar to reigon by dotted line in A demonstrates amyloid-β and tracer deposition patterns within the periarteriolar space. Note that pial membranes (i.e., intimal pia and/or epipia) are absent at this level within intracortical brain, as depicted in summary schematic in lower right-hand side. Note: The vascular smooth muscle cell layer is not depicted in this image. Blue, lectin; cyan/CY5, MeX04; green/FITC, ERTR7; red/Texas Red, TxRd. Scale bars = (A) 10 μm; (B) 5 μm. MeX04 deposition pattern depicted is representative of approximately 500 intracortical vessels from 9 young and old mice.
