# Supplementary material for: Periarteriolar spaces modulate cerebrospinal fluid transport into brain and demonstrate altered morphology in aging and Alzheimer’s disease
Source: Nat Commun. 2022 Jul 6;13:3897. doi: 10.1038/s41467-022-31257-9 (PMC9259669; doi:10.1038/s41467-022-31257-9)
Supplement: Supplementary file 2 — Reporting Summary [file 41467_2022_31257_MOESM2_ESM.pdf]

nature portfolio

Corresponding author(s): Rupal Mehta, MD

Last updated by author(s): May 6, 2022

Reporting Summary

Nature Portfolio wishes to improve the reproducibility of the work that we publish. This form provides structure for consistency and transparency in reporting. For further information on Nature Portfolio policies, see our Editorial Policies and the Editorial Policy Checklist.

Statistics

For all statistical analyses, confirm that the following items are present in the figure legend, table legend, main text, or Methods section.

n/a	Confirmed	
☐	☒	The exact sample size (n) for each experimental group/condition, given as a discrete number and unit of measurement
☐	☒	A statement on whether measurements were taken from distinct samples or whether the same sample was measured repeatedly
☐	☒	The statistical test(s) used AND whether they are one- or two-sided<br>*Only common tests should be described solely by name; describe more complex techniques in the Methods section.*
☒	☐	A description of all covariates tested
☐	☒	A description of any assumptions or corrections, such as tests of normality and adjustment for multiple comparisons
☐	☒	A full description of the statistical parameters including central tendency (e.g. means) or other basic estimates (e.g. regression coefficient) AND variation (e.g. standard deviation) or associated estimates of uncertainty (e.g. confidence intervals)
☐	☒	For null hypothesis testing, the test statistic (e.g. F, t, r) with confidence intervals, effect sizes, degrees of freedom and P value noted<br>*Give P values as exact values whenever suitable.*
☒	☐	For Bayesian analysis, information on the choice of priors and Markov chain Monte Carlo settings
☒	☐	For hierarchical and complex designs, identification of the appropriate level for tests and full reporting of outcomes
☒	☐	Estimates of effect sizes (e.g. Cohen's d, Pearson's r), indicating how they were calculated

Our web collection on statistics for biologists contains articles on many of the points above.

Software and code

Policy information about availability of computer code

Data collection	Nikon Microscope Solutions Imaging Software (NIS-Elements AT Version 4.30.01), Olympus Confocal Microscope Imaging Software (cellSens Version 3 and Oxford Instruments Imaris 3D version 9.5), Abberior Instruments easy 3D STED Microscope Software (Oxford Instruments Imaris 3D Version 9.5), SmartSPIM Light Sheet Fluorescent Microscope Software (Oxford Instruments Imaris 3D version 9.5), Transmission Electron Microscope Software (Digitalmicrograph), and ThorImage®LS version 4.0 (ThorLabs).
Data analysis	Prism 8 (GraphPad Software, Inc, La Jolla, CA), I,ageJ (U.S. National Institutes of Health, Bethesda, MAryland, USA, htt;s://imagej.nih.gov/ij/), Matlab (MathWorks)

For manuscripts utilizing custom algorithms or software that are central to the research but not yet described in published literature, software must be made available to editors and reviewers. We strongly encourage code deposition in a community repository (e.g. GitHub). See the Nature Portfolio guidelines for submitting code & software for further information.

Data

Policy information about availability of data

All manuscripts must include a data availability statement. This statement should provide the following information, where applicable:
- Accession codes, unique identifiers, or web links for publicly available datasets
- A description of any restrictions on data availability
- For clinical datasets or third party data, please ensure that the statement adheres to our policy

Source data are provided with this paper.

Field-specific reporting

Please select the one below that is the best fit for your research. If you are not sure, read the appropriate sections before making your selection.

- [x] Life sciences
- [ ] Behavioural & social sciences
- [ ] Ecological, evolutionary & environmental sciences

For a reference copy of the document with all sections, see nature.com/documents/nr-reporting-summary-flat.pdf

Life sciences study design

All studies must disclose on these points even when the disclosure is negative.

Sample size	Sample size was calculated using a 40% difference between young and old animals with alpha 0.05 and beta 0.20 using G*Power software (Kress et al, Ann Neurol. 2013)
Data exclusions	No data was excluded from this study
Replication	No replicates were conducted in this study. Sample size calculations were performed in order to minimize use of experimental animals and therefore no replicates were done.
Randomization	The study design was not amenable to randomization. The tissue samples from different groups were processed in random order. Individuals performing data abstraction were blinded to the experimental group. Data was then sent to a separate investigator who performed the data analysis, blinded as well. Data was unblinded by investigators only after analysis was completed.
Blinding	Data collection and data analysis were done in a blinded fashion

Reporting for specific materials, systems and methods

We require information from authors about some types of materials, experimental systems and methods used in many studies. Here, indicate whether each material, system or method listed is relevant to your study. If you are not sure if a list item applies to your research, read the appropriate section before selecting a response.

Materials & experimental systems

n/a	Involved in the study	
☐	☒	Antibodies
☒	☐	Eukaryotic cell lines
☒	☐	Palaeontology and archaeology
☐	☒	Animals and other organisms
☒	☐	Human research participants
☒	☐	Clinical data
☒	☐	Dual use research of concern

Methods

n/a	Involved in the study	
☒	☐	ChIP-seq
☒	☐	Flow cytometry
☒	☐	MRI-based neuroimaging

Antibodies

Antibodies used

For validation of antibodies and thin section labeling, three monoclonal rat anti-ERTR7 primary antibodies were used: MA1-40076 (ER-TR7, Invitrogen, Rockford, IL); NB100-64932 (ER-TR7, Novus Biologicals, Centennial, CO); and sc-73355 (ER-TR7, Santa Cruz Biotechnology, Inc., Dallas, TX). Likewise, three mouse or rabbit anti-plectin antibodies were used: sc-33649 (10F6, Santa Cruz Biotechnology, Inc., Dallas, TX); ab32528 (E398P, Abcam, Cambridge, MA); and PA5-79829 (polyclonal, Invitrogen, Rockford, IL). To determine the relationship of ERTR7-positive pial label with afferent cerebral vessels, co-labeling was performed using CY3-conjugated mouse anti-smooth muscle actin antibody (C6198, monoclonal; 1A4, Sigma, St. Louis, MO) and/or rabbit anti-aquaporin 4 antibody (AB3594; polyclonal, Millipore, ab3594, Temecula, CA). Secondary antibody labeling was performed using donkey anti-rat (A21208, Invitrogen, Rockford, IL); donkey anti-mouse (A31570, Invitrogen, Rockford, IL; 715-175-150, Jackson ImmunoResearch Labs, West Grove, PA); and/or donkey anti-rabbit (A31572, Invitrogen, Rockford, IL) antibodies. For thin section labeling, all primary antibodies were used at 1:1000 dilution and all secondary antibodies were used at 1:500 dilution.

Thick sections were sequentially processed using rat anti-ERTR7 (NB100-64932, ER-TR7, Novus Biologicals, Centennial, CO), CY3-conjugated mouse anti-smooth muscle antigen (C6198, 1A4, Sigma, St. Louis, MO), and/or mouse anti-CD68 (MA5-16654, ED1, Invitrogen, Rockford, IL) primary antibodies followed by donkey anti-rat Alexa Fluor 488 (A21208, Invitrogen, Rockford, IL) or donkey anti-mouse CY5 (715-175-150, Jackson ImmunoResearch Labs, West Grove, PA) secondary antibodies. For thick section labeling, all primary antibodies were used at 1:1000 dilution and all secondary antibodies were used at 1:500 dilution.

For cleared samples, the following primary antibodies were used: anti-ERTR7 NB100-64932 (ER-TR7, Novus Biologicals, Centennial, CO) and anti-SMA, MA5-11547 [1A4 (asm-1); Invitrogen, Rockford, IL]. Species appropriate fluorescently conjugated secondary antibodies were applied in 1:2 primary:secondary molar ratios (Jackson ImmunoResearch).

	For immuno-EM, monoclonal rat anti-ERTR7 antibody B100-64932 (Novus Biologicals, Centennial, CO) was used at 1:1200 dilution followed by preadsorbed biotin goat anti-rat secondary antibody (Ab7096, Abcam, Cambridge, UK, 1:200).
Validation	Omission of primary antibodies and rat anti-IgG2a antibody (02-9688; Invitrogen, Rockford, IL) served as negative controls, while lymph node and spleen tissue served as positive controls, and confirmed sensitivity and reproducibility of ERTR7 labels.<br><br>Validated primary antibodies used in the study included:<br><br>Anti ERTR7 (MA1-40076, ER-TR7; Invitrogen, Rockford, IL) validated in: Vaidya A, Mao Z, Tian X, Spencer B, Seluanov A, Gorbunova V. Knock-in reporter mice demonstrate that DNA repair by non-homologous end joining declines with age. PLoS Genet. 201417;10:e1004511. doi: 10.1371/journal.pgen.1004511 [PMID: 25033455; PMCID: PMC4102425].<br><br>Anti ERTR7 (NB100-64932, ER-TR7; Novus Biologicals, Centennial, CO) validated in: Mandl JN, Liou R, Klauschen F et al. Quantification of lymph node transit times reveals differences in antigen surveillance strategies of naive CD4+ and CD8+ T cells Proc Natl Acad Sci 2012;109:18036-41. doi: 10.1073/pnas.1211717109 [PMID: 23071319].<br><br>Anti ERTR7 (sc-73355, ER-TR7; Santa Cruz Biotechnology, Inc., Dallas, TX) validated in: Tostanoski, LH, Chiu YC, Gammon JM, et al. Reprogramming the local lymph node microenvironment promotes tolerance that is systemic and antigen specific. Cell Rep. 16: 2940-2952. doi: 10.1016/j.celrep.2016.08.033 [PMID: 27626664; PMCID: PMC5024722].<br><br>Anti-plectin (sc-33649, 10F6; Santa Cruz Biotechnology, Inc., Dallas, TX) validated in: Laly AC, Sliogeryte K, Pundel OJ, et al. The keratin network of intermediate filaments regulates keratinocyte rigidity sensing and nuclear mechanotransduction. Sci Adv. 2021;7:eabd6187. doi: 10.1126/sciadv.abd6187 [PMID: 33571121; PMCID: PMC7840118].<br><br>Anti-plectin (ab32528, E398P; Abcam, Cambridge, MA) validated in: Croner RS, Sevim M, Metodiev MV, et al. Identification of predictive markers for response to neoadjuvant chemoradiation in rectal carcinomas by proteomic isotope coded protein Label (ICPL) analysis. Int J Mol Sci. 2016;17:209. doi: 10.3390/ijms17020209 [PMID: 26861291; PMC4783941]<br><br>Anti-smooth muscle actin (SMA) antibody (C6198; 1A4; Sigma, St. Louis, MO) validated in: Roholl PJ, Elbers HR, Prinsen I, Claessens JA, van Unnik JA. Distribution of actin isoforms in sarcomas: an immunohistochemical study. Hum Pathol. 1990;21:1269-74. doi: 10.1016/s0046-8177(06)80041-9 [PMID: 2174404].

Animals and other organisms

Policy information about studies involving animals; ARRIVE guidelines recommended for reporting animal research

Laboratory animals	Experiments were performed according to NIH guidelines and protocols were approved by the University of Rochester Committee on Animal Resource (UCAR) (Protocol 2011-023). Mice were housed at ambient temperature of 64-79 degrees Fahrenheit and at humidity of 30-70%. Adult mice (2 months-old, both sexes; C57Bl/6J, Charles River) were used for most experiments. To investigate anatomy of aged mice, double transgenic mice expressing a chimeric mouse/human amyloid precursor protein (Mo/HuAPP695swe) and a mutant human presenilin 1 (PS1-dE9) both directed to CNS neurons were also investigated along with wildtype littermate controls. The APPswe/PS1dE9 (APP/PS1) mice and wildtype controls (13 month-old, both sexes) were purchased from Jackson Laboratory (Bar Harbor, ME, USA).
Wild animals	No wild animals were used in the study.
Field-collected samples	No field collected samples were used in the study.
Ethics oversight	Experiments were performed according to NIH guidelines and protocols approved by the University of Rochester Committee on Animal Resource (UCAR) (Protocol 2011-023).

Note that full information on the approval of the study protocol must also be provided in the manuscript.
