# Supplementary material for: Periarteriolar spaces modulate cerebrospinal fluid transport into brain and demonstrate altered morphology in aging and Alzheimer’s disease
Source: Nat Commun. 2022 Jul 6;13:3897. doi: 10.1038/s41467-022-31257-9 (PMC9259669; doi:10.1038/s41467-022-31257-9)
Supplement: Supplementary file 3 — Description of Additional Supplementary Files [file 41467_2022_31257_MOESM3_ESM.pdf]

Description of Additional Supplementary Files

File name: Supplementary Movie 1

Description: Epipial cell. DAPI/ERTR7, super-resolution image (60X)

File name: Supplementary Movie 2

Description: Large artery, fully ensheathed. DAPI/ERTR7/SMA, confocal image (60X)

File name: Supplementary Movie 3

Description: Small artery, partially ensheathed. DAPI/ERTR7/SMA, confocal image (60X)

File name: Supplementary Movie 4

Description: Cortical mouse brain. Lectin/ERTR7/SMA, CLARITY image (15X)

File name: Supplementary Movie 5

Description: Cortical mouse brain with tracer (30 min). Lectin/ERTR7/TxRd, CLARITY image (15X)

File name: Supplementary Movie 6

Description: Mouse periarteriolar space (Type A) with tracer (30 min). DAPI/ERTR7/TxRd, confocal image (60X)

File name: Supplementary Movie 7

Description: Mouse periarteriolar space (Type B) with tracer (30 min). DAPI/ERTR7/TxRd, confocal image (60X)

File name: Supplementary Movie 8

Description: Mouse periarteriolar space (Type C) with tracer (30 min). DAPI/ERTR7/TxRd, confocal image (60X)

File name: Supplementary Movie 9

Description: Mouse periarteriolar space (Type A) with tracer (30 min). SHG/TxRd, two-photon image (20X)

File name: Supplementary Movie 10

Description: Mouse periarteriolar space (Type B) with tracer (30 min). SHG/TxRd, two-photon image

(20X)

File name: Supplementary Movie 11

Description: Mouse periarteriolar space (Type C) with tracer (30 min). SHG/TxRd, two-photon image (20X)

File name: Supplementary Movie 12

Description: Cortical mouse brain (APP/PS1) with tracer (30 min), coronal PAS section. Lectin/ERTR7MeX04, confocal image (20x)

File name: Supplementary Movie 13

Description: Cortical mouse brain (APP/PS1) with tracer (30 min), deep axial PAS section. Lectin/ERTR7/MeX04, confocal image (20x)
